# C57BL/6J mice best recapitulate fibrosis and inflammatory pathophysiology in syngeneic mouse model of endometriosis

**DOI:** 10.1038/s41598-025-13900-9

**Published:** 2025-08-08

**Authors:** Megha Anchan, Atharvaraj Hande, Samruddhi Deshpande, Richa Patel, Guruprasad Kalthur, Jahnavy Madhukar Joshi, Ratul Datta, Swar Shah, Kriti Sharma, Hiral Pandya, Rahul Dutta

**Affiliations:** 1 Division of Reproductive Biology, Department of Reproductive Science, Kasturba Medical College, Manipal Academy of Higher Education, Manipal, Karnataka 576104 India; 2 Manipal Centre for Biotherapeutics Research, Manipal Academy of Higher Education, Manipal, Karnataka 576104 India; 3 Nova IVF Fertility, Pantaloons Building, Six Mile, Guwahati, Assam 781036 India; 4 Krishna Nursing Home, 4-D, near Delux Bus Stand, Tashkand Society, Nizampura, Vadodara, Gujarat 390002 India; 5 Harsh Hospital & Maternity Home, Surat, Gujarat 395017 India; 6 Sevashram Hospital, Bharuch, Gujarat 392001 India; 7 Center for Animal Research, Ethics & Training, Manipal Academy of Higher Education, Manipal, Karnataka India

**Keywords:** Endometriosis, Animal model, Syngeneic, C57BL/6J, Fibrosis, EMT, Molecular biology, Physiology, Diseases, Medical research

## Abstract

Endometriosis (ENDO), a chronic inflammatory disease affecting approximately 190 million women globally, is characterized by fibrosis, a feature often challenging to replicate in murine models. To identify an optimal syngeneic model exhibiting robust fibrosis and inflammation, we evaluated three inbred mouse strains: C57BL/6J (*n* = 27), BALB/c (*n* = 24), and Swiss albino (*n* = 27). Uterine fragments from donor mice were intraperitoneally transplanted into recipient mice (1 donor: 2 recipients) using an established protocol with minor modifications. All ENDO-induced mice displayed reduced burrowing and exploratory behaviors, alongside increased mechanical hyperalgesia, indicative of ENDO-associated discomfort. Peritoneal fluid analysis revealed a pro-inflammatory environment with a tendency towards an M2 macrophage-dominant profile across all strains. Histological examination confirmed endometriotic lesions with proliferating epithelium (Ki-67+), neovascularization (CD31+), and macrophage infiltration (F4/80+). Notably, C57BL/6J mice exhibited the highest ENDO incidence and a significantly pronounced fibrotic response, evidenced by increased stromal collagen deposition and elevated Col1A1, cytokeratin, α-Smooth Muscle Actin (α-SMA), and Nestin expression. Molecular analysis in C57BL/6J mice further supported epithelial-mesenchymal transition (EMT)-driven fibrosis, with decreased E-cadherin and increased N-cadherin and S100A4 mRNA levels, corroborated by corresponding protein changes (cytokeratin, vimentin, snail). Our findings establish the C57BL/6J strain as the most suitable syngeneic model for ENDO, consistently recapitulating the inflammatory and fibrotic pathophysiology observed in human disease, particularly its fibrotic component.

**Supplementary Information:**

The online version contains supplementary material available at 10.1038/s41598-025-13900-9.

## Introduction

Endometriosis (ENDO) is a prevalent, chronic, estrogen-dependent gynaecological disorder affecting approximately 6–10% (~ 190 million) of reproductive-aged women globally^1^. It is marked by the presence and proliferation of viable ectopic endometrial-like tissue outside the uterus, commonly on the pelvic peritoneum or ovaries^2^. Often simplified as solely dysmenorrhea^3^. ENDO is an etiologically complex, multifaceted inflammatory condition. It is characterized by dysregulated cell proliferation, impaired hormonal signaling, chronic inflammation, immunological dysregulation, angiogenesis, neurogenic inflammation, and notably, epithelial-to-mesenchymal transition (EMT)-induced tissue remodeling and fibrosis^4,5^. ENDO is a major contributor to debilitating chronic pelvic pain (CPP) and infertility. The often underestimated distress of ENDO extends beyond pain, significantly impacting well-being and productivity^6^. Laparoscopically, lesions are classified by distribution, showing significant heterogeneity^7^. Histological diagnosis requires at least two of: endometrial epithelium, glands, stroma, nerve fibers/blood vessels, hemosiderin-laden macrophages, or fibrosis^7^. Despite its commonality, ENDO’s pathogenesis remains unclear, and therapeutic options are limited, emphasizing the need for research^8^.

John Sampson’s 1927 “retrograde menstruation” theory is the most cited explanation for ectopic tissue implantation^9,10^. However, only a subset of women experiencing retrograde menstruation develop ENDO. This suggests additional mechanisms, including immune evasion, implantation, proliferation, peritoneal invasion, and neovascularization^11,12^. The inflammatory microenvironment, rich in alternatively activated macrophages and neutrophils, cytokines, chemokines, and growth factors, is increasingly recognized as a key driver of ENDO^13^. Notably, fibrosis, once viewed as secondary, is now considered a defining characteristic of ENDO^14–17^with growing support for its inclusion in diagnostic criteria^18,19^. The inflammatory milieu in ENDO is closely linked to fibrosis, as indicated by altered immune cell profiles and cytokine levels in peritoneal fluid^20^. Thus, ENDO can be considered both an inflammatory and a fibrotic disease^14^. However, the interplay between inflammation and the development of fibrotic endometriotic implants remains a critical research gap^21^.

Developing effective ENDO therapies necessitates evaluation in animal models that accurately mimic the human disease. However, spontaneous ENDO is limited to menstruating species, with practical limitations^22^. Murine ENDO models are widely used due to their advantages^23^ but rodents do not menstruate, making retrograde menstruation an unlikely mechanism^24^. Consequently, models involve surgical transplantation of uterine tissue or human endometriotic tissue into mice^25–28^ often with estrogen supplementation^26,29^. While human tissue transplantation has benefits, it faces immune rejection issues^30^. Surgical models create lesions resembling clinical ENDO but can disrupt peritoneal immunity^26^. Intraperitoneal injection is also used but may not fully replicate human lesions^31,32^.

Despite advancements in developing rodent models of ENDO, their ability to accurately replicate the progressive fibrosis characteristic of chronic human ENDO remains a significant limitation. Existing models often emphasize the initial formation of lesions and the accompanying immunological responses, largely neglecting the gradual and ongoing progression of fibrosis. This lack of a reliable model that recapitulates the fibrotic progression hinders the investigation of the underlying mechanisms of fibrogenesis and the preclinical evaluation of potential anti-fibrotic therapies. A key challenge lies in the insufficient representation of the progressive fibrotic process observed in human ENDO lesions. Furthermore, current models often provide an inadequate evaluation of key myofibroblast markers such as α-SMA, Collagen I, and Nestin, which are crucial for understanding fibrosis. Comprehensive data on ECM remodeling, with collagen deposition and tissue stiffness in rodents mirroring human fibrotic alterations, are absent. Finally, an over-reliance on in vitro model systems may not fully capture the complex cellular interactions and microenvironmental cues driving fibrosis in vivo. These shortcomings underscore the pressing need for an optimized mouse model that comprehensively replicates the progressive fibrotic aspects of human ENDO.

Therefore, our study aims to establish and validate an experimental fibrotic syngeneic mouse model of ENDO by comparing C57BL/6J, BALB/c, and Swiss albino strains. We seek to identify the strain that most accurately replicates the inflammatory and fibrotic pathophysiology of ENDO by thoroughly characterizing the morphological, histological, and functional features of the generated model. This research provides a comprehensive framework for assessing inflammation and fibrosis in ENDO. We aim to select the optimal mouse strain for accurate disease modeling, addressing significant discrepancies between experimental models and clinical pathology, particularly concerning fibrosis.

##  Methodology

All methods were carried out in accordance with relevant guidelines and regulations. All procedures were performed as described below.

### Ethical approvals

#### Human sample experiments

This study involving human samples was approved by the Institutional Ethics Committee (IEC1: 94/2022), Kasturba Medical College and Kasturba Hospital, Manipal, adhering to the Helsinki Declaration of 1964 and its later amendments. Written informed consent was obtained from all patients. Lesions were collected through laparoscopic excision. While patient hormone status was not a primary focus of this study, standard clinical protocols were followed for all sample collection.

#### Animal experiments

The Institutional Animal Ethics Committee at Kasturba Medical College, Manipal, approved the use of animals (Approval Number: C57BL/6J- IAEC/KMC/88/2024, BALB/c- IAEC/KMC/45/2022, Swiss albino- IAEC/KMC/56/2022). Institutional guidelines and the guidelines of the Committee for the Purpose of Control and Supervision of Experiments on Animals (CPCSEA)were strictly followed for animal handling, and the reporting of animal experiments follows ARRIVE (Animal Research: Reporting of In vivo Experiments) guidelines. The study utilized adult inbred female mice (8–10 weeks, 23 ± 2 g) of three distinct strains: C57BL/6J, BALB/c, and Swiss albino. All the animals were procured from the Central Animal Research Facility, Manipal Academy of Higher Education. Mice were housed (6 per cage) in an environment-controlled setting (21 ± 2 °C, 50–55% humidity, 12–12 h light-dark cycle) at the Central Animal Research Facility, Manipal Academy of Higher Education, with ad libitum access to water and food.

### Preparation of donor and recipient mice

A total of C57BL/6J (*n* = 27), BALB/c (*n* = 24), and Swiss albino (*n* = 27) mice were used for ENDO induction. For each strain, recipient mice were assigned as follows: C57BL/6J (*n* = 14), BALB/c (*n* = 12), and Swiss albino (*n* = 14). Six control mice were included for each strain. The experimental protocol was adapted from a previously published method^33^ with minor modifications. Syngeneic female donor and recipient mice of the three strains were allowed to acclimate for 5 days before the experimental procedures. During this acclimation period, the estrous cycle stages of each animal were monitored using the vaginal lavage method.

### Development of a syngeneic mouse model of endometriosis

**Fig. 1:**
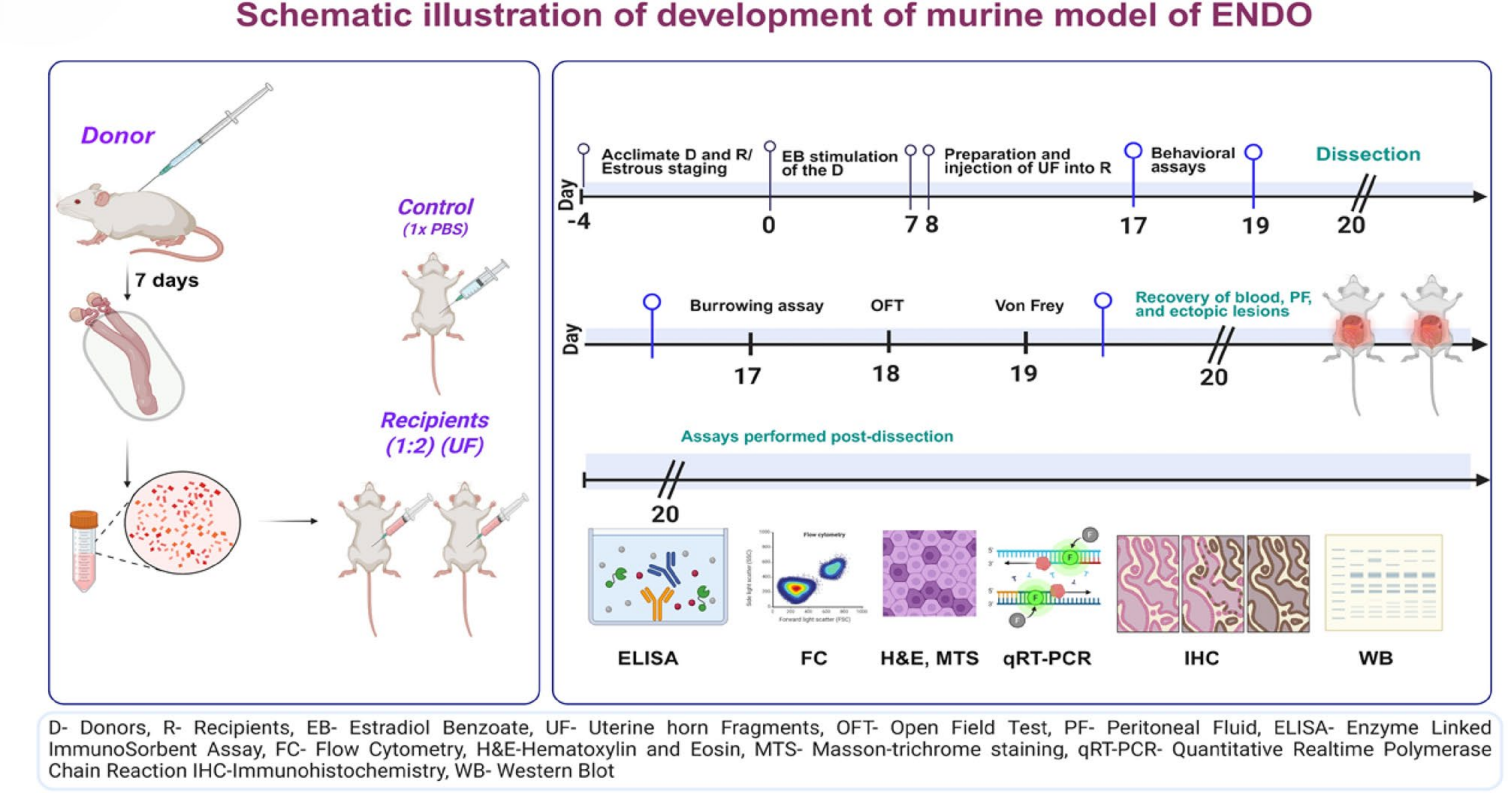
Experimental design and timeline of ENDO induction and behavioral assessments. UH from a single donor mouse were processed into small fragments (~ 1 mm) and equally distributed via I/P injection into two recipient mice to induce endometriosis. Behavioral assessments for pain (burrowing, von Frey) and exploratory behavior (OFT) were conducted between days 16–18 post-induction. Subsequent tissue collection for ELISA, flow cytometry (FC), histology (H&E), Masson’s trichrome staining (MTS), immunohistochemistry (IHC), qRT-PCR, and Western blot (WB) was performed. (Created in part with BioRender.com).

Following confirmation of consistent estrous cycling, donor mice were primed with subcutaneous injections of estradiol benzoate (EB) (TCI chemicals, #E0329) (3 µg/mouse) for seven consecutive days to synchronize their estrous cycles and promote endometrial development. After priming, donor mice were euthanized, and their eutopic uterine horns were harvested. Excess fat and debris were carefully removed, and the uterine horns were rinsed with cold, sterile 1x Phosphate-Buffered Saline (PBS) containing penicillin (100 U/mL) and streptomycin (100 mg/mL) (Pen/strep) (ThermoFisher Scientific #15140122). The uterine tissue was then meticulously minced into small cell aggregation suspensions of uterine fragments (UFs < 0.1 mm) containing both eutopic endometrium and uterine muscle. These UF suspensions were divided into two equal portions, resuspended in 0.5 mL of PBS in a 1 mL syringe (Dispovan), and randomly injected I/P into recipient mice using an 18-gauge needle (0.5 mL per recipient). Thus, each recipient mouse received endometrial tissue from half of a donor uterus. Control mice received subcutaneous injections of estradiol benzoate and I/P injections of 0.5 mL of sterile 1x PBS(without pen/strep). No signs of distress or unusual pain behaviors were observed post-injection. Recipient animals received a single dose of EB before UF injection to synchronize their estrous cycles. Both the recipients and control animals were subsequently administered EB every two days until sacrifice to maintain uniform circulating estrogen levels (as depicted in Fig. 1).

### Behavioral assessment of ENDO mice

Behavioral assays were conducted to assess pain-like behavior and anxiety levels in ENDO-induced mice. All measurements were performed by the same investigator in a blinded manner to ensure consistency and minimize bias. After each test, mice were returned to their home cages.

#### Burrowing assay (assessment of spontaneous behavior)

The burrowing assay, standardized by Deacon with minor modifications^34^ was performed between 16:00 and 18:00 h (3 h before the dark cycle). Control and recipient mice were individually housed in cages equipped with a burrow setup. Mice were initially acclimatized to the burrow tube (20 cm × 6 cm) filled with 200 g of chow diet for 30 min without food in the cage hopper. Water was provided ad libitum. The test was initiated after acclimatization to the laboratory setting with the burrow setup for at least one hour. Each mouse was placed in its cage-burrow setup for two hours. Subsequently, the amount of feed burrowed was measured by weighing the displaced amount in the morning.

#### Electronic von Frey reflex (EvF) (assessment of mechanical hyperalgesia)

Mechanical hyperalgesia was assessed using an electronic von Frey filament approach (Dynamic Plantar Aesthesiometer, Ugo Basile) based on methodologies described by Barrot and Gregory^35,36^. Mice were individually placed in Plexiglas chambers on a wire mesh floor and allowed to acclimate for 1 h until exploratory behavior ceased. A calibrated von Frey filament touch probe (Ugo Basile) applied a gradually increasing force (up to 10 g) to the abdomen. A definite withdrawal response (abdominal retraction, licking, or flinching), along with the corresponding filament force (grams) and duration (seconds), was recorded as the withdrawal threshold. An interval of at least 30 min was maintained between applications.

#### Open field test (OFT) (assessment of exploratory behavior and anxiety)

Exploratory behavior and anxiety levels were assessed 9 days post-UF injection using the OFT^37^. Control and ENDO mice were individually placed in the center of a transparent Plexiglas box (50 cm × 50 cm × 40 cm) with a clean floor. The box was virtually divided into central and peripheral zones. Each mouse was placed in a corner and allowed to explore for 15 min. The number of entries into the central and peripheral zones, as well as the time spent in them, were recorded using a video-tracking system (Logitech HD C930e webcam). Increased anxiety is indicated by more time spent in the peripheral zones and fewer entries into the central zone. The data were analyzed via ANY-maze 64-bit version 7.48 software.

### Collection and processing of biological samples

On day 12 post-induction, all recipient mice were euthanized by cervical dislocation, and blood, peritoneal fluid (PF), and suspected ENDO-like lesions were collected.

Blood and Serum: Blood was collected via cardiac puncture under Ketamine + Xylazine cocktail (0.1mL/20 g mouse wt. IP) anesthesia. Serum was separated by centrifugation at 3000 × g for 10 min at 4 °C and stored at −80 °C for ELISA-based cytokine analysis (IL-6, TNF-α, TGF-β, and Estrogen).

#### Peritoneal fluid (PF)

The PF was collected from the control and the recipient mice in individual tubes collected by peritoneal lavage with 2 mL of sterile 1x PBS. The fluid was treated with RBC lysis buffer(eBioscience™ 1X RBC Lysis Buffer Catalog number 00-4333-57) for 15 min and centrifuged to eliminate erythrocytes. The cells were subsequently resuspended in sterile 1x PBS for flow cytometry analysis of M1 and M2 macrophage populations.

#### Ectopic lesions

Ectopic lesions were photographed for documentation using smart phone camera. Selected lesions were either fixed in Bouin’s solution for 24 h, then transferred to 70% ethanol and embedded in paraffin for histological and immunohistochemical (IHC) analyses, or flash-frozen in liquid nitrogen and stored at −80 °C for RNA (E-cadherin, N-cadherin, and S100A4) and protein (Cytokeratin, Snail, and Vimentin) analyses. Recipient mice without visible lesions were excluded from further investigation.

### Validation of the syngeneic mouse model of ENDO

#### Model success rate

The overall success rate for each strain was calculated as the percentage of recipients with confirmed ectopic lesions. This was done by evaluating recipient mice for ENDO-like lesions based on gross morphology, histological analysis (H&E staining), and the presence of characteristic IHC markers such as Ki67 (proliferation), CD31 (neovascularization), and F4/80 (macrophages).

#### Estrogen ELISA

Serum estradiol (E2) levels were quantified using a commercially available ELISA kit (ELK Biotechnology CO., LTD, #ELK8407) following the manufacturer’s instructions. Estradiol concentrations (pg/mL) were determined by comparing sample absorbance (450 nm) to a standard curve generated with serially diluted estradiol standards, analyzed in duplicate using a microplate reader (MultiSkan FC Microplate Photometer with SkanIt software).

#### Flow cytometry analysis of peritoneal fluid (PF)

PF cells were collected and treated with RBC lysis buffer as described earlier. After washing, 1 × 10^6^ cells were incubated with fluorophore-conjugated antibodies: anti-MO-CD11b-Alexa Fluor 488, anti-MO-CD86-APC, and anti-MO-CD206-PE (all from eBioscience). After incubation, cells were washed and resuspended in PBS. Flow cytometry analysis was performed using a BD Accuri™ C6 Plus flow cytometer, and data were analyzed using FlowJo software to determine the percentage of positive cells for each marker.

### Characterization of ectopic lesions

#### Hematoxylin and eosin (H&E) staining

Ectopic ENDO lesions and control eutopic endometrium were fixed, embedded in paraffin, and sectioned (4 μm). Sections were deparaffinized, rehydrated, stained with hematoxylin (Sigma-Aldrich, #HX03021349) and eosin (Sigma-Aldrich, #1.15935), and mounted with DPX mountant (Sisco Research Laboratories Pvt Ltd, # 88147). Slides were examined using a bright-field microscope (Nikon Eclipse Ei 4 W), and representative images were captured. Histological assessment confirmed the presence of epithelial glands and stromal cells as previously described^38^. Samples not exhibiting endometrial morphology were excluded.

#### Immunohistochemistry (IHC)

Paraffin-embedded ectopic lesions from all three mouse strains were immunostained with primary antibodies against Ki67 (proliferation), CD31 (blood vessels), and F4/80 (macrophages) (all from ThermoFisher Scientific). Briefly, sections were deparaffinized, rehydrated, and antigens were retrieved using sodium citrate buffer (Sigma-Aldrich, Missouri, USA #C7254). Sections were permeabilized with bovine serum albumin (BSA) (HiMedia, India #MB083) in Triton X-100 (Sisco Research Laboratories Pvt Ltd #2024271), and blocked with 5% goat serum (Genei, #163018010A) before overnight incubation with primary antibodies at 4 °C, followed by incubation with appropriate HRP-conjugated secondary antibodies Immunoreactive signals were visualized using 3,3′-Diaminobenzidine (DAB) (Sigmafast, Sigma-Aldrich, #D4293) (Sigma-Aldrich), and sections were counterstained with hematoxylin (Sigma-Aldrich, #HX03021349). Slides were mounted with DPX mountant, and representative images were captured using a Nikon microscope (Nikon Eclipse Ei 4 W, Nikon, Tokyo, Japan) and analyzed using ImageJ software. Antibody details are provided in Table 1.

### Evaluation of fibrotic phenotype

#### Quantification of collagen deposition by Masson-Trichrome staining (MTS)

Collagen deposition in ectopic lesions was assessed using MTS^39^. Sections were fixed, embedded, deparaffinized, and rehydrated. They were then sequentially stained with hematoxylin (Sigma-Aldrich #HX03021349), Biebrich scarlet-acid fuchsin solution(Loba chemie Pvt Ltd, #3855D), phosphomolybdic-phosphotungstic acid(Loba chemie Pvt Ltd, #05265)for 10–15 min, and, and aniline blue (Sisco Research Laboratories Pvt. Ltd.). After dehydration and mounting, slides were examined under a light microscope. The area of collagen deposition (blue staining) was quantified as a proportion of the total ectopic lesion area using ImageJ software.

#### COL1A1 ELISA

The levels of Collagen Type I (COL1A1) in ectopic tissue lysates were quantified using a commercially available ELISA kit (Krishgen Biosystems, USA, #111111111). Lesions were homogenized in 1x PBS, centrifuged at 12,000 × g for 20 min at 4 °C, and the supernatant was collected. ELISA was performed according to the manufacturer’s instructions using 50 µL of lysate per sample, analyzed in duplicate. Optical density was measured at 450 nm, and COL1A1 concentrations were calculated using a standard curve.

#### Quantification of iron deposition by prussian blue staining

Iron deposits in ectopic lesions were identified using Prussian blue staining based on Perls’ reaction^40^. Sections were treated with a freshly prepared mixture of 5% potassium ferrocyanide (Sigma Aldrich #244023) and hydrochloric acid (v/v), counterstained with nuclear fast red^41^ dehydrated, and mounted. Images were captured using a Nikon microscope, and the area of iron deposition (blue staining) was measured using ImageJ software (https://imagej.nih.gov/ij/download.html, RRID: SCR_003070).

#### Immunostaining for EMT markers

Epithelial-to-mesenchymal transition (EMT) status in ENDO lesions was assessed by immunostaining for Cytokeratin (epithelial marker), α-Smooth Muscle Actin (α-SMA, mesenchymal/fibrotic marker), and Nestin (marker of cellular plasticity/intermediate mesenchymal state) (all from Cell Signaling Technologies or ThermoFisher Scientific; details in Table 1). The immunostaining procedure was performed as described previously.

**Table 1:** Antibodies used in the study primary antibodies.

Primary antibodies					
Target	Primary antibody	Species raised in	Dilution used (IHC/WB)	Manufacturer & catalog number	RRID
Proliferating cells (Ki67)	Anti-Ki67 (Monoclonal)	Rat	1:100 IHC	ThermoFisher Scientific, #14-5698-82	AB_10854564
Blood vessels (CD31/PECAM)	Anti-CD31 (Monoclonal)	Rabbit	1:100 IHC	ThermoFisher Scientific, #14-0311-81	AB_467201
Macrophages (F4/80)	Anti-F4/80 (Monoclonal)	Rat	1:50 IHC	ThermoFisher Scientific, #14-4801-82	AB_467558
Pan-keratin (C11) mouse mAb	Pan-keratin (Monoclonal)	Mouse	1:500 IHC 1:1000 WB	Cell Signaling TECHNOLOGIES #4545	-
αSMA	Anti- αSMA (Monoclonal)	Mouse	1:200 IHC	ThermoFisher Scientific, #14-976080	AB_2572996
Nestin	Anti- αSMA (Monoclonal)	Mouse	1:200 IHC	ThermoFisher Scientific, #14-584380	AB_1907436
Vimentin rabbit pAb	Vimentin (Polyclonal)	Rabbit	1:1000 WB	ABclonal #A11952	AB_2861643
Snail rabbit pAb	Snail (Polyclonal)	Rabbit	1:1000 WB	ABclonal #A5243	AB_2766076

Secondary antibodies for DAB IHC					
Target	Secondary antibody	Species raised in	Dilution used	Manufacturer & catalog number	RRID
Rat IgG (H + L)	Goat anti-rat IgG (H + L) (HRP-conjugated)	Goat	1:1000 IHC	ThermoFisher Scientific, #A18865	AB_2535642
Mouse IgG Fc	Goat anti-Mouse IgG Fc Secondary Antibody, HRP	Goat	1:1000 IHC 1:10000 WB	ThermoFisher Scientific, #A16084	AB_2534758
Rabbit IgG Fc	Goat anti-Rabbit IgG Fc Secondary Antibody, HRP	Goat	1:1000 IHC 1:10000 WB	ThermoFisher Scientific, #A16116	AB_2534789

Antibodies used for flow cytometry					
Target	Primary antibody	Species raised in	Dilution used	Manufacturer & catalog number	RRID
CD 11b Mouse	Anti-Mo-CD 11b-Alexa flour 488 Monoclonal	Rat	0.5 µg/test	eBioscience # 53-0112-80	AB_469901
CD 86 Mouse	CD86 (B7-2) monoclonal antibody (GL1), APC-eFluor™ 780	Rat	0.06 µg/test	eBioscience # 17-0862-81	AB_469418
CD 206 Mouse	Anti-Mo-Cd206 (MMR) Monoclonal Antibody (MR6F3), PE	Rat	0.125 µg/test	eBioscience # 12-2061-80	AB_2637422
CD 68 Mouse	Anti-Mo-CD68 (FA-11), PE	Rat	0.25 μg/test	eBioscience #12-0681-80	AB_2572569

### Measurement of pro-inflammatory cytokines

Snap-frozen serum samples were thawed, and cytokine concentrations (IL-6 (ABclonal, #RK00008), TNF-α (ABclonal, #RK00027), and TGF-β (ABclonal, #RP01458), were measured using mouse-specific ELISA kits according to the manufacturer’s instructions. Serum from control mice was used to establish baseline cytokine levels. Standard curves were generated for each cytokine, and samples were analyzed (*n* = 6 per group). The detection limits for IL-6, TNF-α, and TGF-β were 7.2, 6.5, and 3.9 pg/mL, respectively.

### RNA extraction and quantitative real-time PCR (qRT-PCR)

Total RNA was extracted from frozen ENDO lesions (*n* = 3 biological replicates) using a Qiagen RNeasy Mini Kit (Qiagen, #74104). Complementary DNA (cDNA) was synthesized using the PrimeScript RT Reagent Kit (TaKaRa Bio Inc., #RR037A). qRT-PCR was performed using Sybr^®^ Premix Ex Taq™ II (Tli RNase H Plus, TakaraBio, #RR820A) on a StepOne real-time PCR system (Applied Biosystems) to assess the gene expression of fibrotic markers: E-cadherin, N-cadherin, and S100A4, with GAPDH as an internal control. Primer efficiencies and specificities were confirmed to be between 90% and 110%. Reactions were performed in duplicate, and amplification included initial denaturation (98 °C, 2 min) followed by 40 cycles of denaturation (98 °C, 30 s), annealing (optimal temperature, 30 s), and extension (72 °C, 45 s). All samples were examined in triplicate. Primer sequences were custom-synthesized by Bioserve Biotechnologies (India) Pvt Ltd and are provided in Table 2.

**Table 2:** Primer sequences used in the experiments.

Gene	Oligonucleotide sequence (5′ to 3′)	Product size (bp)
GAPDH	F- ATGGGACGATGCTGGTACTGA R- TGCTGACAACCTTGAGTGAAAT	117
E-cadherin	F- AACCCAAGCACGTATCAGGG R- ACTGCTGGTCAGGATCGTTG	142
N-cadherin	F- CACTGCCATTGATGCGGATG R- TGCCACAGTGATGATGTCCC	136
S100A4	F- TTGTGGTTGAGCTGTGGGAG R- GGTAACCGTTGAGACCCCTC	122

### Western blot analysis

Protein profiling for fibrosis-associated markers was performed on ectopic lesions by western blotting. Snap-frozen ectopic lesions and corresponding eutopic endometrium were homogenized in RIPA lysis buffer with protease inhibitor cocktail (TCI chemicals, #P2976). Equivalent amounts of protein (30 µg) were separated by SDS-PAGE (10–12%) and blotted onto PVDF membranes. Membranes were blocked with 5% non-fat dry milk and incubated overnight at 4 °C with primary antibodies against Cytokeratin (ABclonal, #A5243), Snail (ABclonal, #A5243), and Vimentin (ABclonal, #A11952). After washing with TBST, membranes were incubated with appropriate secondary antibodies (1:10000) at RT for 1 h. Immunoreactive bands were visualized using enhanced chemiluminescence and a gel documentation system (GE healthCare Systems, Amersham Imager 600).

## Statistical analysis

Statistical analysis was performed using GraphPad Prism (version 10.2). Data from two groups were analyzed using Student’s t-test (unpaired, two-tailed, 95% CI, significance defined as *p* < 0.05). Ordinary one-way ANOVA was used for comparisons of three or more groups. Data are presented as mean ± standard error of the mean (SEM) of triplicate measurements. Significant differences are indicated by asterisks in figures (* *p* < 0.05, ** *p* < 0.01, *** *p* < 0.001, **** *p* < 0.0001).

## Results

### ENDO mice exhibit behavioral alterations suggestive of pain and anxiety

**Fig. 2:**
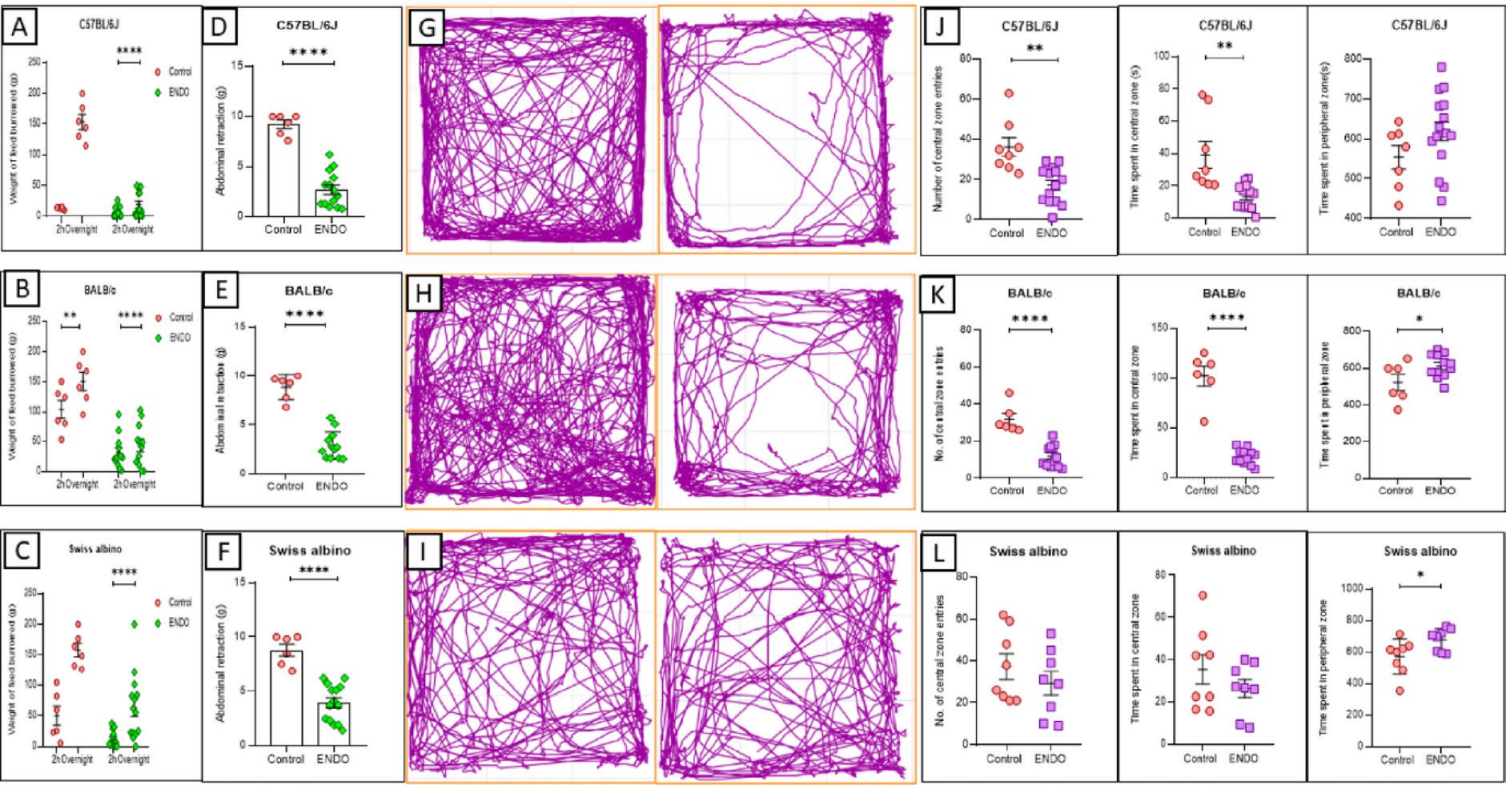
(**A**) C57BL/6J ENDO mice showed significantly reduced burrowing activity, heightened mechanical sensitivity (Von Frey), and altered Open Field Test (OFT) parameters (decreased central zone duration/entries, increased peripheral zone time) compared to controls, indicative of pain-related behavior. (**B**) BALB/c ENDO mice also exhibited significant impairment in burrowing, increased Von Frey sensitivity, and reduced central zone activity in OFT, suggesting persistent pain or anxiety-like behavior. (**C**) Swiss albino ENDO mice displayed diminished burrowing, heightened mechanical sensitivity, and decreased central zone activity in OFT, consistent with pain-related behavioral modifications observed in other strains. While no significant strain-specific differences were observed, BALB/c and C57BL/6J mice showed a tendency towards a more pronounced burrowing deficit. (*n* = 6 per control group, *n* = 14 per ENDO group for C57BL/6J and Swiss albino, *n* = 12 per ENDO group for BALB/c; ***P < 0.001, ****P < 0.0001). Trajectory plots for C57BL/6J (**G**), BALB/c (**H**), and Swiss albino (**I**) ENDO and control mice in the Open Field Test, visually confirming diminished overall movement in ENDO mice.(**D**, **E**, **F**) Significantly heightened mechanical hypersensitivity in ENDO mice of all strains compared to their respective control groups. (**J**, **K**, **L**) Statistically significant decrease in the number of entries into the central and peripheral zones, a shorter duration of time spent in the central area, and increased time spent in the peripheral zones in the ENDO groups compared to the controls.

The burrowing assay, a measure of well-being and potential pain-related behavior, revealed a significant reduction in the amount of chow displaced by ENDO mice compared to their respective controls across all strains. Specifically, C57BL/6J ENDO mice displaced an average of 5.79 ± 7.30 g after 2 h and 19.02 ± 19.43 g overnight, significantly less than the control group (2 h: 12.9 ± 1.66 g; overnight: 153.38 ± 28.30 g) (Fig. 2A). Similarly, BALB/c ENDO mice showed a substantial decrease in burrowing, with an average displacement of 31.94 ± 26.13 g at 2 h and 43.61 ± 32.93 g overnight, compared to controls (2 h: 103.99 ± 33.32 g; overnight: 150.63 ± 34.90 g)(Fig. 2B). Swiss albino ENDO mice also exhibited reduced burrowing, displacing 12.94 ± 12.02 g at 2 h and 63.52 ± 51.92 g overnight, compared to controls (2 h: 50.13 ± 35.22 g; overnight: 157.67 ± 63.52 g) (Fig. 2C).

Mechanical hypersensitivity, assessed using the electronic von Frey test, was significantly heightened in ENDO mice of all strains compared to their respective control groups (Fig. 2D, E, F). This was evidenced by a markedly lower mechanical threshold (force in grams required to elicit a withdrawal response in ENDO mice, indicating an increased sensitivity to tactile abdominal stimulation.

In the OFT, used to evaluate exploratory behavior and anxiety levels, ENDO mice across all strains exhibited reduced exploratory activity. The trajectory plots (Fig. 2G, H, I) visually confirmed the diminished overall movement in ENDO mice. This is indicated by a statistically significant decrease in the number of entries into the central and peripheral zones, as well as a shorter duration of time spent in the central, more exposed area of the arena (Fig. 2J, K, L). Swiss albino mice showed similar trends but without statistical significance for central zone entries or time spent. The increased time spent in the peripheral zones further indicates heightened anxiety-like behavior in the ENDO groups, though this was not statistically significant in C57BL/6J mice.

### Successful establishment of a syngeneic mouse model of ENDO with high incidence in C57BL/6J mice

**Fig. 3:**
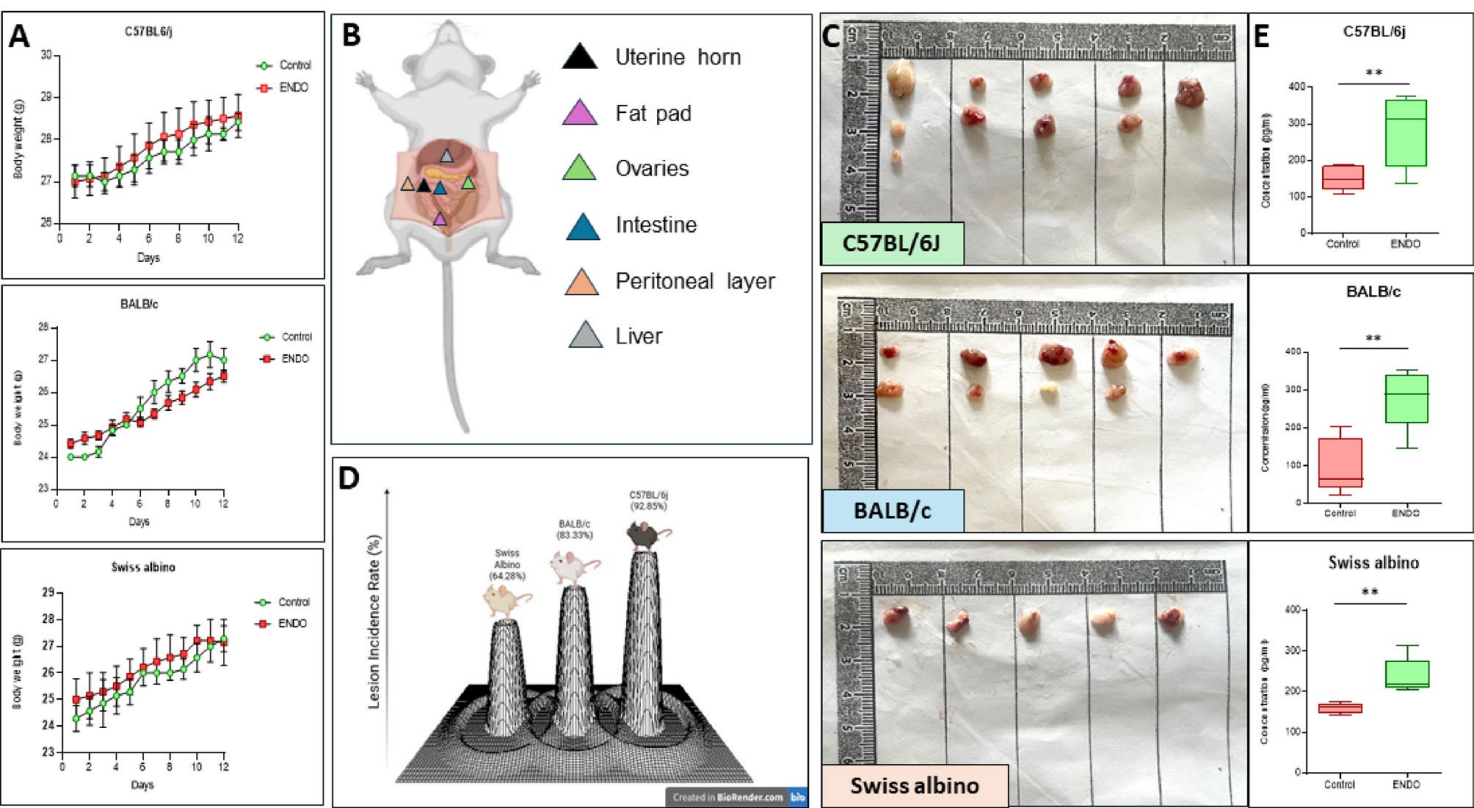
General health, lesion distribution, incidence, and estrogen levels in the endometriosis mouse model. (**A**) Body Weight Monitoring: Cumulative mean ± SEM body weight of control and ENDO mice post-injection. No significant differences (*P* > 0.05) were observed between groups throughout the experimental period, indicating that ENDO induction did not compromise the general well-being of recipient mice despite a transient minor decrease in the ENDO group after day 7. (**B**) Ectopic Lesion Distribution: Representative illustration showing the common anatomical locations of ectopic lesions identified 12 days post-ENDO induction across all mouse strains, including adipose tissue, peritoneal layer, uterine horn, ovaries (adhesions), and internal organs (intestine). (**C**) Macroscopic Appearance of Ectopic Lesions: Representative in situ photographs of ectopic lesions observed across the three mouse strains. Lesions typically presented as superficial, white cystic or red nodules/lesions exhibiting varying degrees of inflammation. Control mice injected with 1× PBS did not develop any lesions. (**D**) Incidence of Endometriosis Across Strains: The incidence of ENDO varied significantly among strains, with C57BL/6J exhibiting the highest success rate (92.85%, 13/14), followed by BALB/c (83.33%, 10/12) and Swiss albino (64.28%, 9/14). (**E**) Circulating Estrogen Levels: ELISA analysis revealed significantly increased circulating estrogen levels in ENDO mice compared to their respective controls across all three strains (*p* < 0.01, unpaired t-test), indicating systemic estrogen dependence and hormonal dysregulation associated with the model. Data are presented as mean ± SEM.

I/P injection of minced donor uterine horn tissue fragments into the recipient animals was well-tolerated, with no significant mortality or adverse effects on the overall health and body weight of the recipient mice (Fig. 3A). This resulted in the successful development of ectopic lesions across all three inbred strains after 12 days (Fig. 3B). Macroscopic examination at necropsy revealed the presence of multiple ectopic lesions in all ENDO-induced mice, varying in size (small to moderate) and morphology (cystic or solid) with diverse coloration (white, brown, red, or black) (Fig. 3C). The lesions were predominantly on peritoneal surfaces, adipose tissue, and pelvic organs.

The prevalence of endometriosis, defined as the percentage of recipient mice exhibiting visible ectopic lesions, demonstrated a significant strain-dependent variation (Fig. 3D). The C57BL/6J strain showed the highest incidence at 92.85% (13 out of 14 mice), followed by BALB/c at 83.33% (10 out of 12 mice), and Swiss albino at 64.28% (9 out of 14 mice). Circulating serum estrogen levels, measured by ELISA, were significantly (*p* < 0.01) elevated in ENDO mice compared to their respective sham controls across all three strains, confirming the estrogen-dependent nature of the induced lesions (Fig. 3E). To account for the impact of estrogen priming, the control animal animals were injected with EB every alternate day until sacrifice.

### Histopathological analysis confirms typical endometriotic lesion morphology

**Fig. 4:**
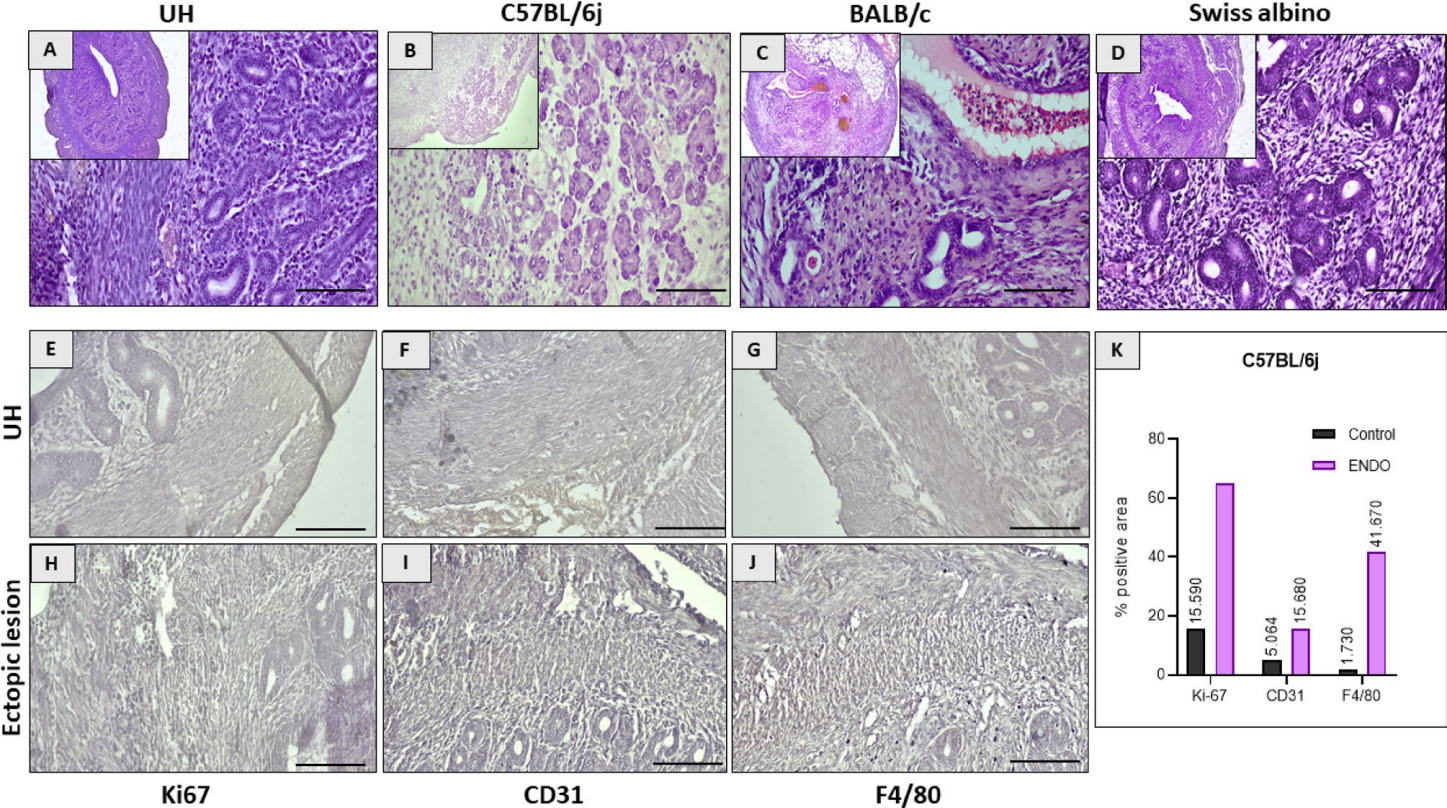
Histological confirmation and cellular characterization of ectopic endometriotic lesions. (**A**) Control uterine horn (UH) from healthy mice in the estrous stage displayed normal morphology (H&E, *n* = 6). (**B**–**D**) Ectopic lesions harvested at day 12 post-induction from C57BL/6J, BALB/c, and Swiss albino mice (H&E, *n* = 6 per group) exhibited characteristic endometriotic features, including singular or multi-layered epithelium, dense immune cell infiltration in the stroma, and the presence of endometrial glands. (**E**–**G**) Immunohistochemical analysis of control uterus sections (**E**–**G**, Control) revealed (**H**) proliferating epithelial cells (Ki67+), (**I**) established vasculature (CD31/PECAM1+), and (**J**) a significant presence of macrophages (F4/80+). Representative images for (**H**, **I**, **J**) are shown from C57BL/6J ectopic lesions, and similar staining patterns were observed across all three strains. (**K**) Quantitative analysis of IHC staining across all ENDO lesions from all strains (*n* = 3 per group) indicated a high proliferative index (65.18% Ki67+), substantial vascularization (15.68% CD31+), and marked macrophage infiltration (41.67% F4/80+). (Magnification and scale bars to be consistently applied throughout the figure. Example: Magnification = 4x, scale bar = 40 μm; Magnification = 40x, Scale bar = 10 μm).

Histological evaluation of the ectopic lesions using H&E staining confirmed the presence of characteristic endometriotic features in all three strains (Fig. 4B–D). These included the presence of endometrial glands with surrounding stroma, evidence of previous microhemorrhages indicated by hemosiderin-laden macrophages within the stroma, and the formation of new blood vessels. These histological features were consistent with the expected morphology of endometriotic lesions and resembled the structure of the control UH obtained from mice in the estrous phase (Fig. 4A).

IHC analysis of the lesions further characterized their cellular composition. Compared to the uterus (Fig. 4E), there was a significant increase in the number of Ki67-positive cells in the ectopic lesions(Fig. 4H), indicating enhanced cell proliferation. Similarly, compared to the control (Fig. 4F), the density of CD31-positive endothelial cells, marking neovascularization, was significantly higher in the lesions (Fig. 4I). Furthermore, relative to the control (Fig. 4G) a substantial infiltration of F4/80-positive macrophages was evident in the stromal regions of the ectopic lesions across all strains (Fig. 4J). Across all ENDO lesions analyzed by quantitative IHC (Fig. 4K), we observed a high level of cell proliferation (65.18% Ki67+), significant vascularization (15.68% CD31+), and a notable presence of macrophages (41.67% F4/80+). While all strains exhibited these markers, visual inspection suggested potentially higher levels in C57BL/6J lesions, consistent with its heightened fibrotic and inflammatory profile.

### Strain-dependent dysregulation of inflammation and macrophage polarization

**Fig. 5:**
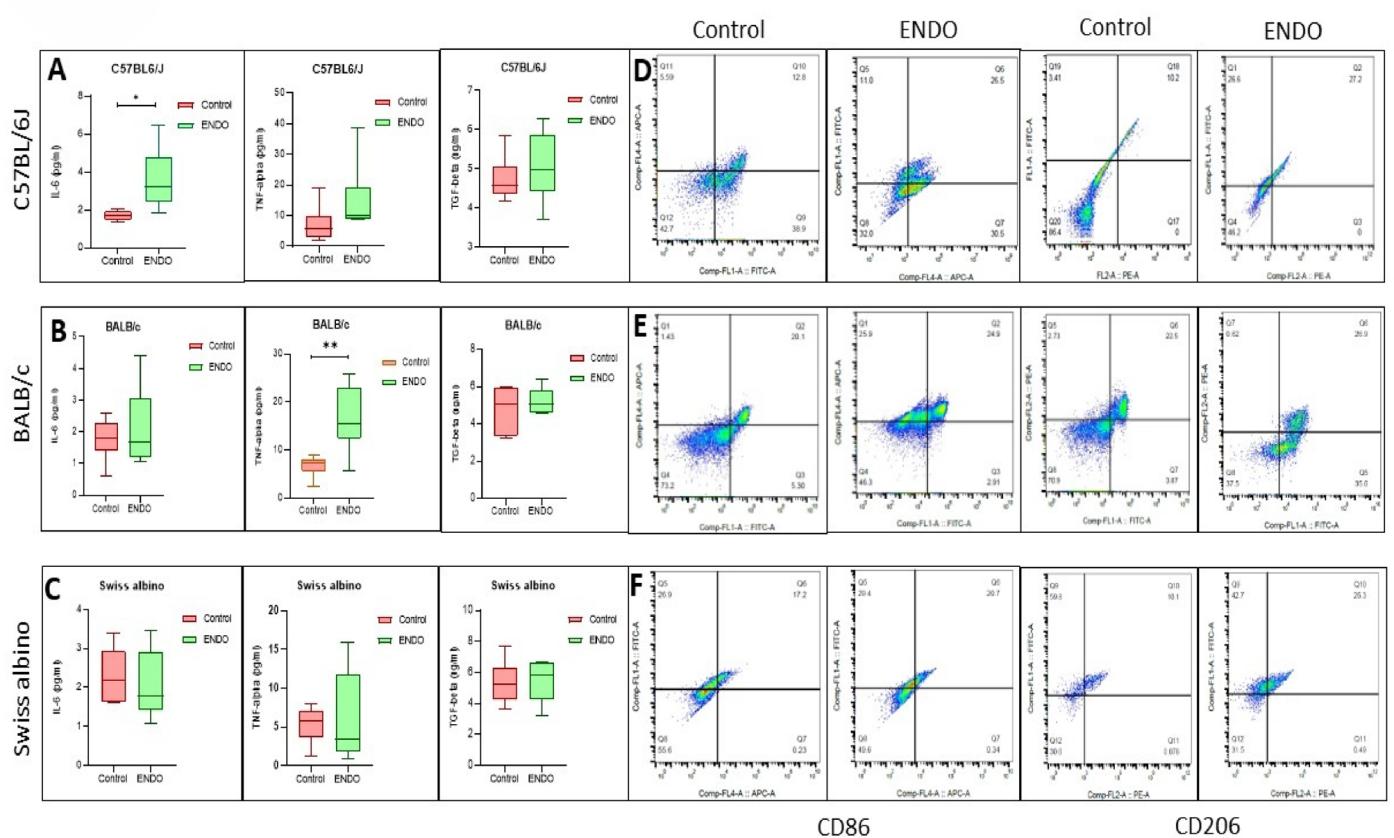
Strain-dependent systemic inflammation and peritoneal macrophage polarization in endometriosis. (**A**) Serum cytokine analysis in C57BL/6J ENDO mice revealed a significant increase in IL-6, with modest elevations in TNF-α and TGF-β. (**B**) BALB/c ENDO mice showed a prominent rise in TNF-α, accompanied by minor increases in IL-6 and TGF-β. (**C**) In contrast, Swiss albino ENDO mice exhibited no significant alterations in circulating levels of these cytokines. (**D**–**F**) Peritoneal fluid flow cytometry demonstrated the percentage of M1 (CD11b + CD86+) and M2 (CD11b + CD206+) macrophages in ENDO mice of each strain: (**D**) C57BL/6J showed a balanced M1/M2 population (26.5% vs. 27.2%), (**E**) BALB/c displayed similar M1 (24.9%) and M2 (26.9%) percentages, and (**F**) Swiss albino exhibited M1 (20.7%) and M2 (25.3%) populations.

Analysis of circulating pro-inflammatory cytokines in serum samples revealed a distinct strain-specific inflammatory response to ENDO induction (Fig. 5A–C). C57BL/6J ENDO mice exhibited a marked and statistically significant elevation in IL-6 levels compared to controls, with modest but non-significant increases in TNF-α and TGF-β. In BALB/c ENDO mice, TNF-α levels were significantly increased compared to controls, with slight, non-significant increases in IL-6 and TGF-β. In contrast, Swiss albino ENDO mice did not show any statistically significant alterations in the circulating levels of these three cytokines.

Flow cytometric analysis of peritoneal fluid macrophages revealed strain-specific patterns of M1 (CD86⁺CD11⁺) and M2 (CD206⁺CD11⁺) macrophage populations (Fig. 5D–F). In the C57BL/6J strain, M1 and M2 macrophages were present at 26.5% and 27.2% of the CD11b + population, respectively, indicating a balanced and robust immune cell involvement. BALB/c mice showed M1 and M2 populations at 24.9% and 26.9%, respectively, suggesting a similarly active but slightly more M2-skewed profile. Swiss albino mice exhibited M1 and M2 populations at 20.7% and 25.3%, respectively, indicating a comparatively lower overall macrophage activation in the peritoneal fluid. These percentages represent average values from flow cytometry analysis across multiple mice for each strain (*n* = 3 for C57BL/6J, Swiss albino, and BALB/c). While M2 populations were numerically higher than M1 populations across all strains, suggesting a pro-fibrotic environment, statistical significance for this difference was not performed in this study.

### Ectopic lesions display increased fibrosis markers and iron deposition

**Fig. 6:**
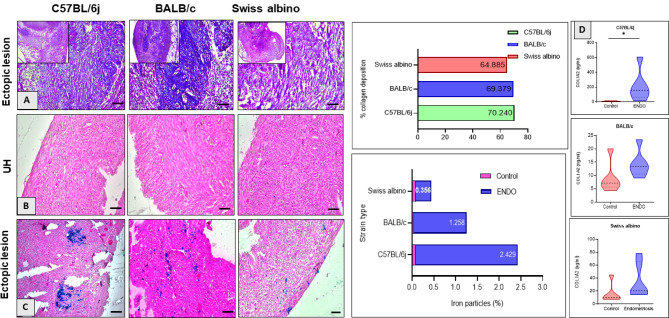
Assessment of fibrosis and iron accumulation in ectopic lesions across strains. (**A**) Masson’s trichrome staining (MTS) revealed varying degrees of collagen deposition (blue) in ectopic lesions, with C57BL/6J exhibiting the most pronounced fibrosis, followed by substantial fibrosis in BALB/c (quantified at 69.95%), and the least in Swiss albino (57.47%). (**B**, **C**) Prussian blue staining showed strain-dependent iron deposition (blue precipitates, arrowheads) in the lesion stroma, with significant accumulation in C57BL/6J (score: 2.348), moderate in BALB/c (score: 1.208), and minimal in Swiss albino (score: 0.356, OD: 0.084). (**D**) Collagen Type I alpha 1 (Col1A1) ELISA indicated significant stromal fibrosis in C57BL/6J (*p < 0.05), moderate fibrosis in BALB/c (lower than C57BL/6J but higher than Swiss albino), and mild fibrosis in Swiss albino. Data are mean ± SEM, *p < 0.05 (unpaired t-test).

Masson’s trichrome staining, used to visualize collagen deposition, demonstrated a substantial presence of collagen-rich fibrotic areas (blue staining) in the stromal regions of ectopic lesions across all three strains (Fig. 6A). Quantitative analysis revealed a significant increase in the collagen-positive area in C57BL/6J ENDO mice compared to controls and the other strains, indicating more pronounced fibrotic remodeling. While the numerical difference in percentage between C57BL/6J (69.95%) and BALB/c (69.05%) was small, C57BL/6J exhibited a statistically significant difference in collagen deposition when compared to Swiss albino (57.47%) and controls, indicating more pronounced fibrotic remodeling in C57BL/6J. Prussian blue staining revealed a considerable accumulation of iron deposits (blue staining) within the stroma of the ectopic lesions in all strains (Fig. 6C) compared to the control horn(Fig. 6B), suggesting chronic microhemorrhaging and inflammation within the lesions. ELISA of ectopic lesion lysates confirmed significantly elevated protein levels of Col1A1 in the lesions compared to control uterine tissues across all strains. The highest levels of this key component of the fibrotic extracellular matrix were observed in C57BL/6J mice (Fig. 6D).

### C57BL/6J ENDO lesions exhibit pronounced EMT and fibrotic marker expression resembling human ENDO 

**Fig. 7:**
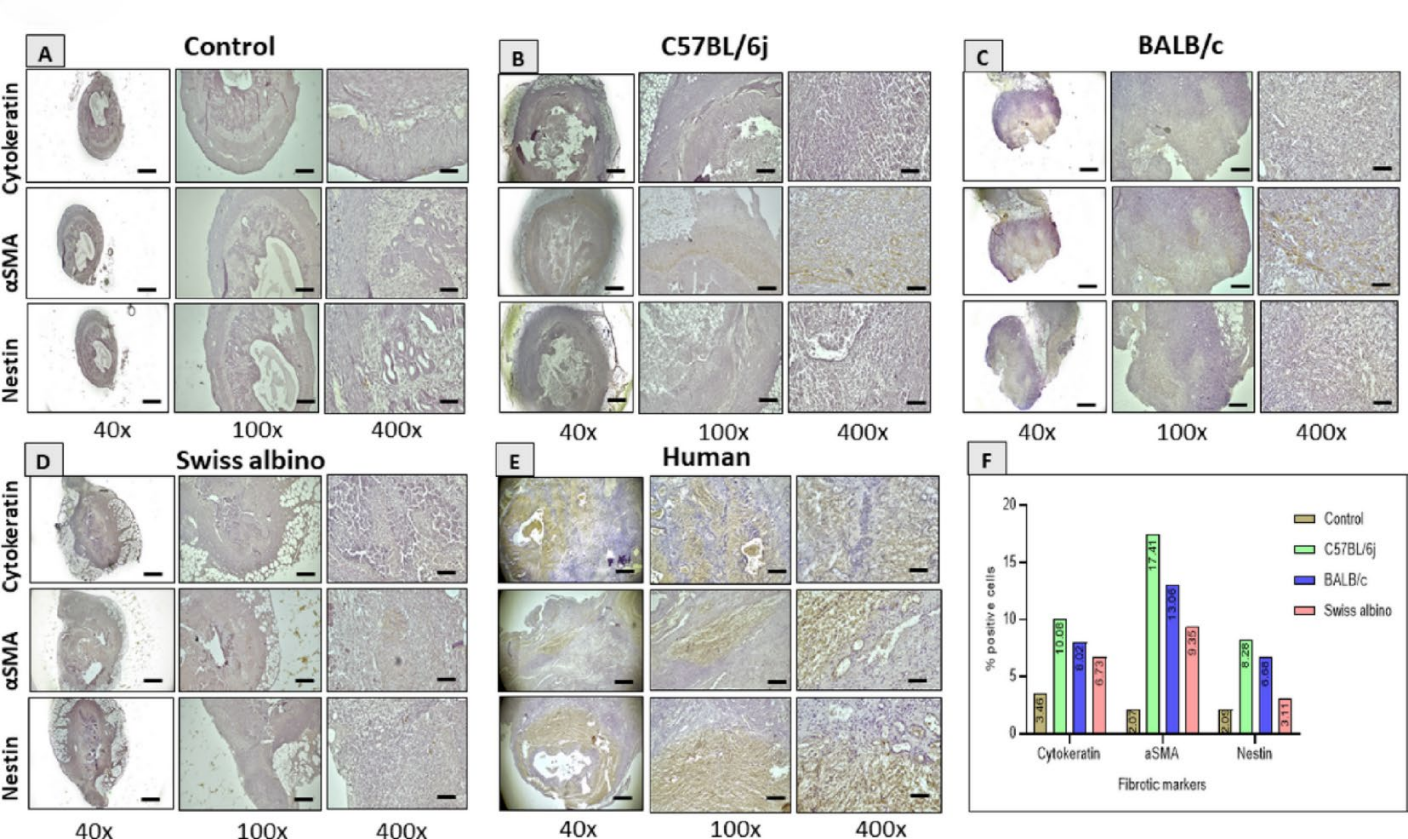
Strain-dependent expression of fibrotic markers in ectopic lesions and translational relevance to human endometriosis. (**A**, **E**) Control tissues exhibited negligible immunohistochemical staining for α-SMA, Nestin, and Cytokeratin (quantified as low percentages). (**B**, **E**) C57BL/6J ectopic lesions displayed the most pronounced fibrotic profile, characterized by the highest percentages of α-SMA, Nestin, and Cytokeratin positive cells. (**C**, **E**) BALB/c lesions showed intermediate expression levels of these markers, while (**D**, **E**) Swiss albino lesions exhibited the lowest expression. Data are presented as the percentage of positively stained cells across the tissue field. (**E**) Comparative IHC analysis of Cytokeratin, Vimentin, and Nestin in ectopic lesions from the mouse model and human endometriosis samples revealed similar patterns of marker localization and staining intensity, supporting the model’s translational significance. (DAB brown, Hematoxylin blue counterstain; Magnification: 40x, 100x, 1000x). (**F**) Percentage of positively stained cells for all three markers (α-SMA: 17.41%, Nestin: 8.28%, Cytokeratin: 10.08%.

Immunohistochemical analysis of fibrotic and EMT-related markers (α-SMA, Nestin, Cytokeratin) revealed minimal expression in control uterine tissues (Fig. 7A). In contrast, C57BL/6J lesions showed strong expression of these markers (Fig. 7B). BALB/c and Swiss albino lesions showed moderate and lower expression levels, respectively (Fig. 7C, D). Notably, the staining patterns observed in our mouse model, particularly in C57BL/6J mice, qualitatively resembled those seen in human ENDO tissue (Fig. 7E), suggesting potential translational relevance, though this comparison was not quantitatively analyzed for statistical significance. C57BL/6J lesions exhibited the highest percentage of positively stained cells for all three markers (α-SMA: 17.41%, Nestin: 8.28%, Cytokeratin: 10.08%), indicative of a significant fibrotic and EMT signature. Lesions used for immunostaining were collected at day 12 post-induction and represent various morphologies (cystic or solid) observed across the strains. Figure 7F represents the average percentage of positively stained cells across the tissue field from multiple mice for each strain (*n* = 3 per group for IHC analysis).

**Fig. 8:**
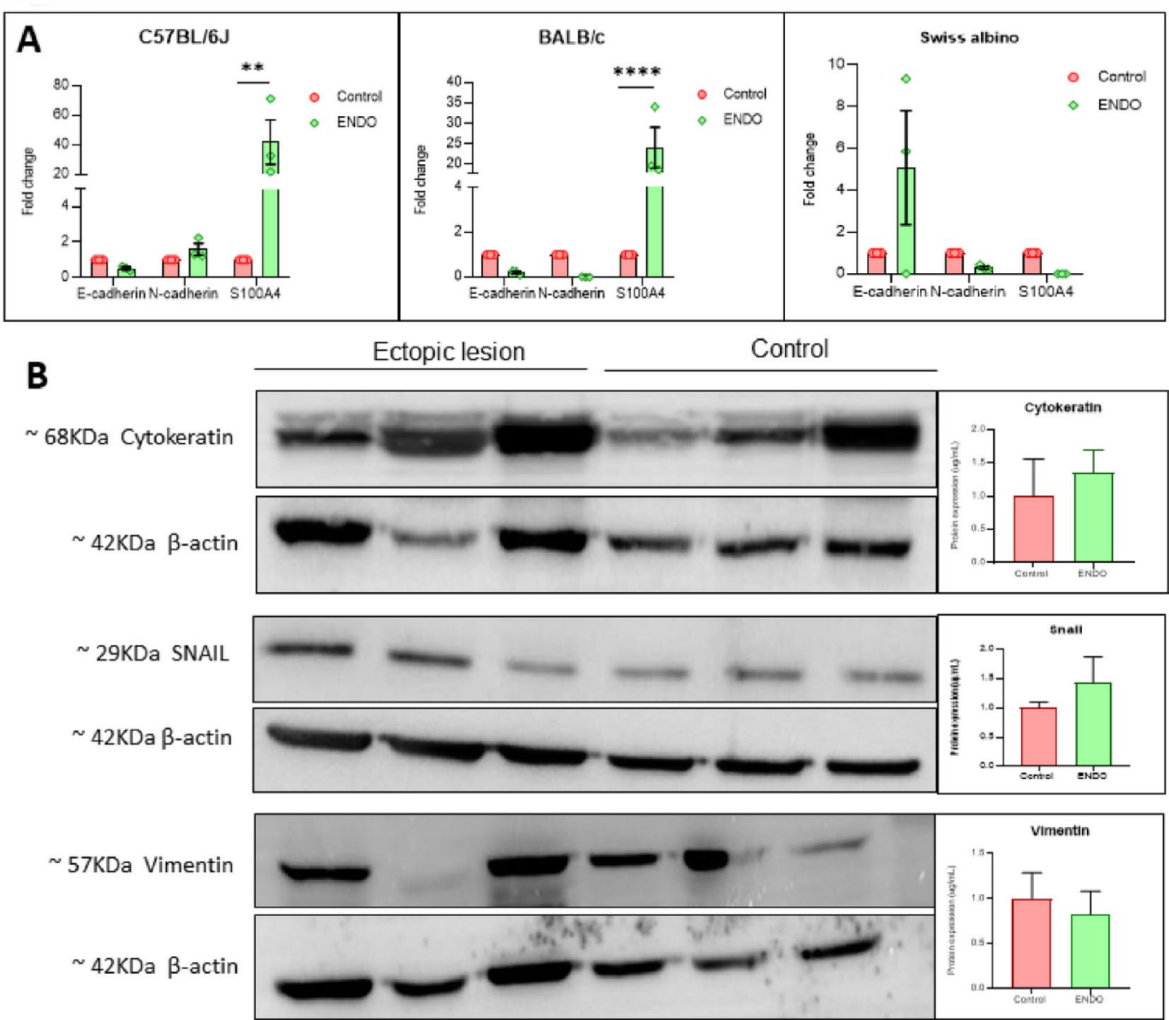
Differential EMT Marker expression at the transcriptional and translational levels in endometriotic lesions. (**A**) mRNA expression profiles of E-cadherin, N-cadherin, and S100A4 in ectopic lesions across three mouse strains. C57BL/6J lesions displayed a robust EMT signature with E-cadherin downregulation and significant upregulation of N-cadherin and S100A4 (**P < 0.01, ****P < 0.0001). BALB/c lesions showed evidence of partial EMT with reduced E-cadherin, low N-cadherin, and moderate S100A4. Swiss albino lesions exhibited inconsistent E-cadherin levels and minimal mesenchymal marker expression. (**B**) Western blot analysis in C57BL/6J mice (*n* = 3 per group) revealed increased protein levels of Snail (ENDO: 1.43 µg/mL, Control: 0.99 µg/mL) and Cytokeratin (ENDO: 1.35, Control: 0.98) in ENDO lesions, while Vimentin expression was reduced (ENDO: 0.82 µg/mL, Control: 1.11 µg/mL).

Gene expression analysis using qRT-PCR revealed a classic EMT profile in C57BL/6J lesions, with a consistent downregulation of E-cadherin (0.52-fold change) and significant upregulation of mesenchymal markers N-cadherin (1.59-fold change) and S100A4 (42.66-fold change) (Fig. 8A).BALB/c lesions also showed evidence of partial EMT, with a significant upregulation of S100A4, alongside reduced E-cadherin and low N-cadherin. Swiss albino lesions exhibited inconsistent E-cadherin levels and minimal mesenchymal marker expression. To validate mRNA expression patterns of C57BL/6j, protein levels of cytokeratin, snail, and vimentin were assessed through densitometric analysis of immunoreactive bands in ENDO lesions and corresponding control tissues from C57BL/6J mice (*n* = 3 per group). Snail expression was numerically higher in ENDO lesions (mean = 1.43 µg/mL) than in controls (mean = 0.99 µg/mL), suggesting the transcriptional activation of EMT pathways. Cytokeratin expression was also numerically elevated in lesions (mean = 1.35 µg/mL), indicating retention of epithelial characteristics. Vimentin levels were numerically diminished in ENDO lesions (mean = 0.82 µg/mL) relative to controls, potentially suggesting a potential divergence from the traditional EMT state (Fig. 8B). These observations, while indicative of trends, did not reach statistical significance in this analysis.

## Discussion

Our study comprehensively characterizes a syngeneic mouse model of endometriosis across three commonly used inbred strains: C57BL/6J, BALB/c, and Swiss albino. We meticulously compared lesion development, behavioral alterations indicative of pain and anxiety, the inflammatory milieu, macrophage polarization, and the progression of fibrosis. The findings suggest the C57BL/6J strain as the most promising model for recapitulating the inflammatory and fibrotic pathophysiology of human ENDO, particularly its fibrotic component. This model was established through a straightforward, cost-effective, and short intraperitoneal injection protocol. The model effectively mimics key aspects of the human disease, like collagen deposition, pain-related behavioral changes, and the upregulation of crucial EMT biomarkers.

The behavioral assays provided compelling evidence for the development of pain and anxiety-like behaviors in the ENDO mice. Significant reduction in burrowing activity was observed across all strains. This aligns with findings in other animal models and symptomatic women with ENDO, where discomfort and pain often lead to decreased engagement^42^. The heightened mechanical hypersensitivity observed in the ENDO mice further supports the presence of endometriosis-associated hyperalgesia, a hallmark of the human condition^43^. The diminished exploratory behavior and increased time spent in the peripheral zones of the OFT suggest elevated anxiety levels, which are frequently comorbid with chronic pelvic pain in women with ENDO^23^. The C57BL/6J strain consistently exhibited the most pronounced behavioral alterations, making it suitable for investigating the mechanisms underlying ENDO-associated pain.

The successful induction of ectopic lesions in all ENDO-challenged mice, coupled with the absence of significant morbidity, underscores the feasibility and safety of our syngeneic model^31^. The elevated circulating estrogen levels were observed in all ENDO groups. This corroborates the well-established estrogen-dependent nature of endometriosis^29^ and validates the hormonal milieu supporting lesion development in our model. The significantly higher prevalence of lesion formation in the C57BL/6J strain points towards inherent differences in immune responses or hormonal sensitivity.

Histopathological analysis confirmed that the induced lesions in all strains displayed typical glandular and stromal components as observed in human ENDO^44,45^. There was increased cell proliferation, neovascularization, and macrophage infiltration within the lesions. This is characteristic of human endometriotic implants, highlighting the active and dynamic nature of these ectopic tissues^46,47^. The heightened angiogenic and macrophage infiltration observed in C57BL/6J lesions suggests a more active inflammatory and fibrotic environment in this strain.

The strain-dependent differences in systemic inflammation and macrophage polarization are noteworthy. The marked elevation of IL-6 in C57BL/6J mice and TNF-α in BALB/c mice suggests distinct inflammatory pathways are activated in response to ENDO in these strains^48,49^. The increased M2 macrophage population in the peritoneal fluid of all ENDO strains aligns with previous findings, indicating the pro-fibrotic environment of endometriosis^50^. The most dramatic alterations in pro-fibrotic immune subsets observed in C57BL/6J mice are consistent with its heightened fibrotic and inflammatory profile^51^.

A key finding of our study is the significant increase in collagen deposition and Col1A1 protein levels in the ectopic lesions, particularly in the C57BL/6J strain. This robust fibrotic response in C57BL/6J mice mimics the excessive collagen deposition that defines fibrotic ENDO in humans^17,18^. The substantial iron deposits in the lesions further indicate chronic microhemorrhage and inflammation. Iron overload is known to contribute to fibrosis in various tissues^52,53^.

The immunohistochemical and molecular analyses of EMT and fibrotic markers provide strong evidence for the involvement of EMT in the fibrotic remodeling. The lesions in C57BL/6J showed high expression of α-SMA, Nestin, and Cytokeratin, resembling the patterns observed in human ENDO tissue. This underscores the activation of myofibroblasts and the ongoing tissue remodeling^54^. The gene expression data, showing a classical EMT signature in C57BL/6J mice (downregulated E-cadherin, upregulated N-cadherin, and S100A4), further supports this notion. The Western blot findings in C57BL/6J lesions showed elevated Snail and Cytokeratin and decreased Vimentin. This suggests a dynamic and potentially complex EMT process, warranting further investigation to fully understand its role in the fibrotic progression of endometriosis^55^.

This study provides a comprehensive characterization of syngeneic mouse models for endometriosis, but we acknowledge several limitations. Firstly, murine models do not naturally menstruate, making the widely accepted theory of retrograde menstruation not replicable in mice. The current model relies on the injection of uterine fragments, which may not fully mimic the complex clinical processes leading to human lesion establishment. Secondly, we have compared mouse lesions to human tissue samples to provide qualitative visual validation. This aspect was not quantitatively analyzed for statistical significance, therefore, direct translational comparability may be missing. Future studies using RNA sequencing or advanced proteomics for in-depth comparative analysis between mouse strains and human samples would provide further insights into the molecular similarities and differences. Additionally, while behavioral assays like burrowing used for the study are not specific to endometriosis-associated pain alone. Estrogen and inflammation are the two most prominent characteristics in endometriosis. In this work, we did not study the interplay of estrogen and inflammatory pathways. Future research could explore the local estrogen metabolism within the lesions.

**Fig. 9:**
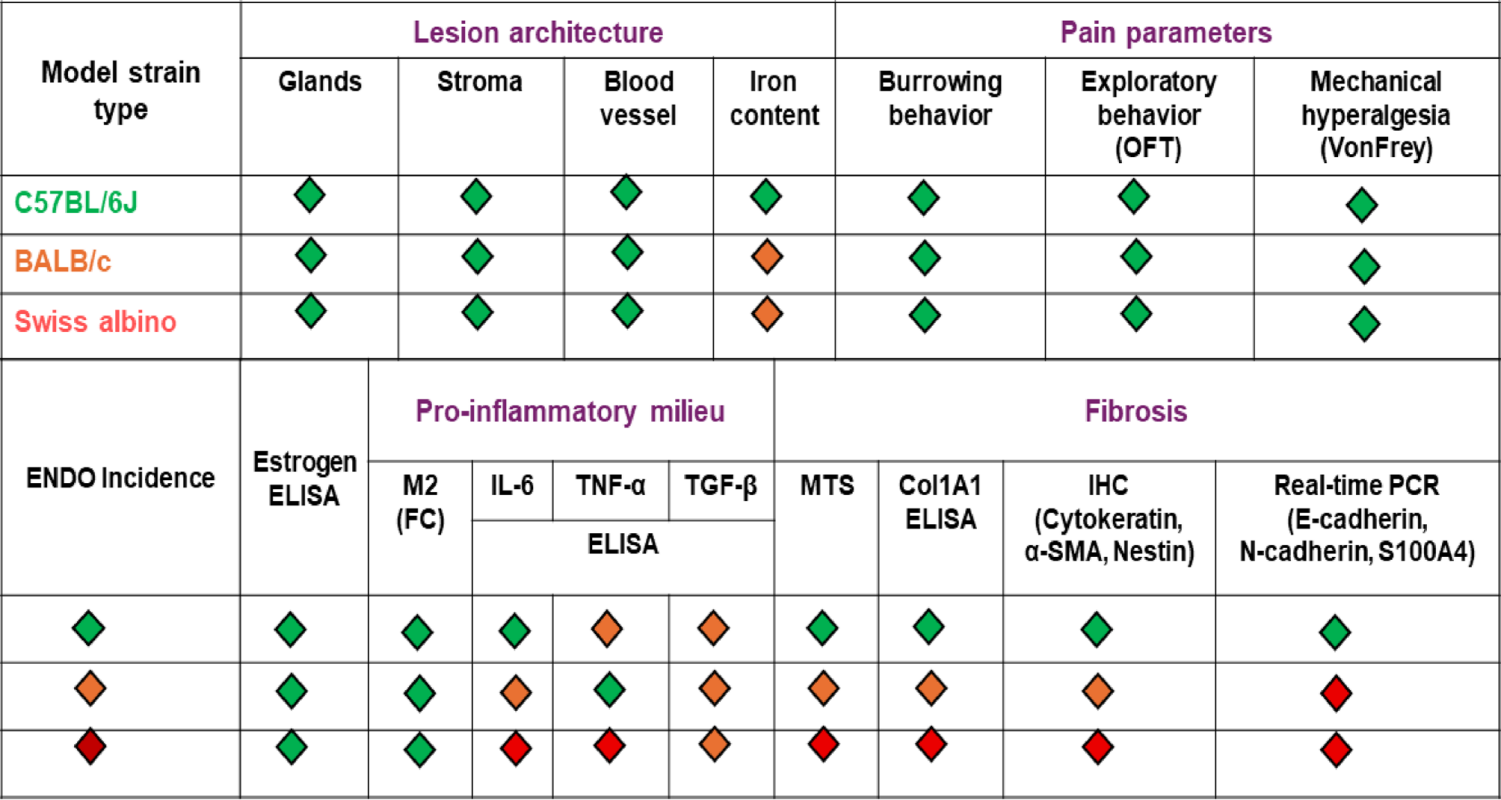
Comparative performance of mouse strains in modeling syngeneic endometriosis. A traffic light rating system (Dorning et al. 2021) was used to compare the ability of C57BL/6J, BALB/c, and Swiss albino mice to model key characteristics of syngeneic endometriosis. Green indicates excellent, orange moderate, and red poor performance across defined parameters.

In conclusion, our comprehensive comparison of three inbred mouse strains demonstrates that the C57BL/6J syngeneic model most accurately recapitulates the key pathological features of human endometriosis. The model showed behavioral alterations indicative of pain and anxiety, a pro-inflammatory and M2 macrophage-skewed immune environment, and a robust fibrotic response driven by EMT-related mechanisms. This model offers a significant ability to mimic the progressive fibrosis, a critical aspect of chronic ENDO. The C57BL/6J model is promising for future research. aimed at understanding molecular pathways of fibrosis in endometriosis and for the preclinical evaluation of novel anti-fibrotic strategies^56^. Using a traffic light-based classification^57^ C57BL/6J receives the maximum green lights, indicating confirmation of the clinical phenotype (Fig. 9). BALB/c comes promising second in this classification. However, the Swiss albino strain showed inconsistent results across various parameters, making it unsuitable for ENDO modelling. Further investigations into strain-dependent variations observed in our study are warranted to enhance our understanding of endometriosis pathogenesis and to facilitate the development of more effective treatments^57,58^.

## Supplementary Information

Below is the link to the electronic supplementary material.

Supplementary Material 1

## Data Availability

The original contributions presented in the study are included in the article; further inquiries can be directed to the corresponding author.

## References

[1] Leone, R. et al. Epidemiology of infertility in women with endometriosis. *Best Practi. Res. Clin. Obstet. Gynaecol.* **92**, 102454 (2024). 10.1016/j.bpobgyn.2023.102454 38183767

[2] Sherwani, S. et al. The vicious cycle of chronic endometriosis and depression-an immunological and physiological perspective. *Front. Med.* **11**, 1425691 (2024). 39309679 10.3389/fmed.2024.1425691 PMC11412830

[3] Fleming, A. & Hardy, A. Endometriosis is more than a painful period. Period. *J. Nurse Pract.* **21**, 105232 (2025).

[4] Bouic, P. J. Endometriosis and infertility: The hidden link between endometritis, hormonal imbalances and immune dysfunctions preventing implantation! *JBRA Assist. Reprod.* **27**, 144–146 (2023). 37348006 10.5935/1518-0557.20230015 PMC10279451

[5] Mosleh, H. et al. Role of neuropeptides in patients with endometriosis: A literature review. *Middle East. Fertil. Soc. J.* **29**, 49 (2024).

[6] Della Corte, L. et al. The burden of endometriosis on women’s lifespan: A narrative overview on quality of life and psychosocial wellbeing. *Int. J. Environ. Res. Public Health* **17**, 683 (2020). 32610665 10.3390/ijerph17134683 PMC7370081

[7] Camboni, A. & Marbaix, E. Ectopic endometrium: The pathologist’s perspective. *Int. J. Mol. Sci.* **22**, 10974 (2021). 34681634 10.3390/ijms222010974 PMC8540175

[8] Mehedintu, C., Plotogea, M. N., Ionescu, S. & Antonovici, M. Endometriosis still a challenge. *J. Med. Life* **7**, 349–357 (2014). 25408753 PMC4233437

[9] Sampson, J. A. Metastatic or embolic endometriosis, due to the menstrual dissemination of endometrial tissue into the venous circulation. *Am. J. Pathol.* **3**, 93–110 (1927). 19969738 PMC1931779

[10] Vercellini, P., Viganò, P., Somigliana, E. & Fedele, L. Endometriosis: Pathogenesis and treatment. *Nat. Rev. Endocrinol.* **10**, 261–275 (2014). 24366116 10.1038/nrendo.2013.255

[11] Yuan, M. et al. Rediscovering peritoneal macrophages in a murine endometriosis model. *Hum. Reprod.* **32**, 94–102 (2017). 27816922 10.1093/humrep/dew274

[12] Laschke, M. W. & Menger, M. D. Basic mechanisms of vascularization in endometriosis and their clinical implications. *Hum. Reprod. Update* **24**, 207–224 (2018). 29377994 10.1093/humupd/dmy001

[13] Monnin, N., Fattet, A. J. & Koscinski, I. Endometriosis update of pathophysiology, (Epi) genetic and environmental involvement. *Biomedicines* **11**, 978 (2023). 36979957 10.3390/biomedicines11030978 PMC10046867

[14] Viganò, P. et al. Cellular components contributing to fibrosis in endometriosis: A literature review. *J. Minim. Invasive Gynecol.* **27**, 287–295 (2020). 31785417 10.1016/j.jmig.2019.11.011

[15] Burney, R. O. Fibrosis as a molecular hallmark of endometriosis pathophysiology. *Fertil. Steril.* **118**, 203–204 (2022). 35624047 10.1016/j.fertnstert.2022.05.004

[16] Garcia Garcia, J. M. et al. Endometriosis: Cellular and molecular mechanisms leading to fibrosis. *Reprod. Sci.* **30**, 1453–1461 (2023). 36289173 10.1007/s43032-022-01083-x PMC10160154

[17] Vissers, G., Giacomozzi, M., Verdurmen, W., Peek, R. & Nap, A. The role of fibrosis in endometriosis: A systematic review. *Hum. Reprod. Update* **30**, 706–750 (2024). 39067455 10.1093/humupd/dmae023 PMC11532625

[18] Guo, S. W. Fibrogenesis resulting from Cyclic bleeding: The holy Grail of the natural history of ectopic endometrium. *Hum. Reprod.* **33**, 353–356 (2018). 29420711 10.1093/humrep/dey015

[19] Vigano, P. et al. Time to redefine endometriosis including its pro-fibrotic nature. *Hum. Reprod.* **33**, 347–352 (2018). 29206943 10.1093/humrep/dex354

[20] Abramiuk, M. et al. The role of the immune system in the development of endometriosis. *Cells* **11**, 2028 (2022). 35805112 10.3390/cells11132028 PMC9265783

[21] Izumi, G. et al. Involvement of immune cells in the pathogenesis of endometriosis. *J. Obstet. Gynaecol. Res.* **44**, 191–198 (2018). 29316073 10.1111/jog.13559

[22] Anchan, M. M. et al. Unveiling the fibrotic puzzle of endometriosis: An overlooked concern calling for prompt action. *F1000Res* **13**, 721 (2024). 39669683 10.12688/f1000research.152368.3 PMC11635194

[23] Laganà, A. S. et al. Translational animal models for endometriosis research: A long and windy road. *Ann. Transl. Med.* **6**, 431 (2018). 30596061 10.21037/atm.2018.08.24 PMC6281523

[24] Burns, K. A. et al. Endometriosis in the mouse: Challenges and progress toward a ‘best fit’ murine model. *Front. Physiol.* **12**, 806574 (2021). 35095566 10.3389/fphys.2021.806574 PMC8794744

[25] Rossi, G. et al. Dynamic aspects of endometriosis in a mouse model through analysis of implantation and progression. *Arch. Gynecol. Obstet.* **263**, 102–107 (2000). 10763836 10.1007/s004040050005

[26] He, Y. et al. Re-evaluation of mouse models of endometriosis for pathological and immunological research. *Front. Immunol.* **13**, 986202 (2022). 36466829 10.3389/fimmu.2022.986202 PMC9716019

[27] Pelch, K. E. et al. Aberrant gene expression profile in a mouse model of endometriosis mirrors that observed in women. *Fertil. Steril.* **93**, 1615–1627 (2010). 19473656 10.1016/j.fertnstert.2009.03.086 PMC2904074

[28] Greaves, E. et al. A novel mouse model of endometriosis mimics human phenotype and reveals insights into the inflammatory contribution of shed endometrium. *Am. J. Pathol.* **184**, 1930–1939 (2014). 24910298 10.1016/j.ajpath.2014.03.011 PMC4076466

[29] Burns, K. A. et al. Role of estrogen receptor signaling required for endometriosis-like lesion establishment in a mouse model. *Endocrinology* **153**, 3960–3971 (2012). 22700766 10.1210/en.2012-1294 PMC3404357

[30] Brännström, M. et al. Uterus transplantation: From research, through human trials and into the future. *Hum. Reprod. Update* **29**, 521–544 (2023). 37328434 10.1093/humupd/dmad012 PMC10477946

[31] Greaves, E., Critchley, H. O. D., Horne, A. W. & Saunders, P. T. K. Relevant human tissue resources and laboratory models for use in endometriosis research. *Acta Obstet. Gynecol. Scand.* **96**, 644–658 (2017). 28233896 10.1111/aogs.13119 PMC5485163

[32] Tejada, M. A. et al. Rodent animal models of endometriosis-Associated pain: Unmet needs and resources available for improving translational research in endometriosis. *Int. J. Mol. Sci.* **24**, 2422 (2023). 36768741 10.3390/ijms24032422 PMC9917069

[33] Bacci, M. et al. Macrophages are alternatively activated in patients with endometriosis and required for growth and vascularization of lesions in a mouse model of disease. *Am. J. Pathol.* **175**, 547–556 (2009). 19574425 10.2353/ajpath.2009.081011 PMC2716955

[34] Deacon, R. M. J. Burrowing in rodents: A sensitive method for detecting behavioral dysfunction. *Nat. Protoc.* **1**, 118–121 (2006). 17406222 10.1038/nprot.2006.19

[35] Barrot, M. Tests and models of nociception and pain in rodents. *Neuroscience* **211**, 39–50 (2012). 22244975 10.1016/j.neuroscience.2011.12.041

[36] Greaves, E. et al. EP2 receptor antagonism reduces peripheral and central hyperalgesia in a preclinical mouse model of endometriosis. *Sci. Rep.* **7**, 44169 (2017). 28281561 10.1038/srep44169 PMC5345039

[37] Li, T. et al. Endometriosis alters brain electrophysiology, gene expression and increases pain sensitization, anxiety, and depression in female mice. *Biol. Reprod.* **99**, 349–359 (2018). 29425272 10.1093/biolre/ioy035 PMC6692844

[38] Maddern, J. et al. A syngeneic inoculation mouse model of endometriosis that develops multiple comorbid visceral and cutaneous pain like behaviours. *Pain* **163**, 1622–1635 (2022). 35050959 10.1097/j.pain.0000000000002552

[39] Wang, X. et al. The matrix stiffness is increased in the eutopic endometrium of adenomyosis patients: A study based on atomic force microscopy and histochemistry. *Eur. J. Histochem.* **68**, 4131 (2024). 39629520 10.4081/ejh.2024.4131 PMC11694501

[40] Perls, M. Nachweis von Eisenoxyd in Gewissen pigmenten. *Archiv für Pathologische Anatomie und Physiologie und für Klinische Medicin* **39**, 42–48 (1867).

[41] Van Langendonckt, A., Casanas-Roux, F. & Donnez, J. Iron overload in the peritoneal cavity of women with pelvic endometriosis. *Fertil. Steril.* **78**, 712–718 (2002). 12372445 10.1016/s0015-0282(02)03346-0

[42] Dai, Y. et al. Dysmenorrhea pattern in adolescences informing adult endometriosis. *BMC Public Health* **24**, 373 (2024). 38317119 10.1186/s12889-024-17825-2 PMC10840152

[43] Odagiri, K. et al. Smooth muscle metaplasia and innervation in interstitium of endometriotic lesions related to pain. *Fertil. Steril.* **92**, 1525–1531 (2009). 18930216 10.1016/j.fertnstert.2008.08.101

[44] Gordts, S., Koninckx, P. & Brosens, I. Pathogenesis of deep endometriosis. *Fertil. Steril.* **108**, 872–885e1 (2017). 29100623 10.1016/j.fertnstert.2017.08.036

[45] Moen, M. H. & Halvorsen, T. B. Histologic confirmation of endometriosis in different peritoneal lesions. *Acta Obstet. Gynecol. Scand.* **71**, 337–342 (1992). 1326207 10.3109/00016349209021069

[46] Asante, A. & Taylor, R. N. Endometriosis: The role of neuroangiogenesis. *Annu. Rev. Physiol.* **73**, 163–182 (2011). 21054165 10.1146/annurev-physiol-012110-142158

[47] Rocha, A. L. L., Reis, F. M. & Taylor, R. N. Angiogenesis and endometriosis. *Obstet. Gynecol. Int.* **2013**, 859619 (2013). 10.1155/2013/859619 PMC3677669 23766765

[48] Braun, K. M. & Diamond, M. P. The biology of adhesion formation in the peritoneal cavity. *Semin Pediatr. Surg.* **23**, 336–343 (2014). 25459438 10.1053/j.sempedsurg.2014.06.004

[49] Song, S. Y. et al. Endometriosis-related chronic pelvic pain. *Biomedicines* **11**, 2868 (2023). 37893241 10.3390/biomedicines11102868 PMC10603876

[50] Ono, Y. et al. CD206 + macrophage is an accelerator of endometriotic-like lesion via promoting angiogenesis in the endometriosis mouse model. *Sci. Rep.* **11**, 853 (2021). 33441630 10.1038/s41598-020-79578-3 PMC7807007

[51] Jiang, Y. et al. Macrophages in organ fibrosis: From pathogenesis to therapeutic targets. *Cell. Death Discov.* **10**, 487 (2024). 39632841 10.1038/s41420-024-02247-1 PMC11618518

[52] Burns, K. A. et al. Early endometriosis in females is directed by immune-mediated estrogen receptor α and IL-6 Cross-Talk. *Endocrinology* **159**, 103–118 (2018). 28927243 10.1210/en.2017-00562 PMC5761597

[53] Mehta, K. J., Farnaud, S. J. & Sharp, P. A. Iron and liver fibrosis: Mechanistic and clinical aspects. *World J. Gastroenterol.* **25**, 521–538 (2019). 30774269 10.3748/wjg.v25.i5.521 PMC6371002

[54] Baez, P., Ruiz, A., Colon, M., Salvo, V. A. & Flores, I. SNAIL regulates E-Cadherin expression in human endometrial cells. *Biol. Reprod.* **81**, 340 (2009).

[55] Hjelmeland, M. E. et al. Loss of vimentin expression in preoperative biopsies independently predicts poor prognosis, lymph node metastasis and recurrence in endometrial cancer. *BJC Rep.* **2**, 1–9 (2024). 10.1038/s44276-024-00105-2 PMC11524127 39516342

[56] Perrone, U. et al. A review of phase II and III drugs for the treatment and management of endometriosis. *Expert Opin. Emerg. Drugs* **28**, 333–351 (2023). 38099328 10.1080/14728214.2023.2296080

[57] Dorning, A. et al. Bioluminescent imaging in induced mouse models of endometriosis reveals differences in four model variations. *Dis. Model. Mech.* **14**, dmm049070 (2021). 34382636 10.1242/dmm.049070 PMC8419713

[58] Mori, T. et al. Local Estrogen formation and its regulation in endometriosis. *Reprod. Med. Biol.* **18**, 305–311 (2019). 31607790 10.1002/rmb2.12285 PMC6780031

