# Supplementary material for: C57BL/6J mice best recapitulate fibrosis and inflammatory pathophysiology in syngeneic mouse model of endometriosis
Source: Sci Rep. 2025 Aug 8;15:29024. doi: 10.1038/s41598-025-13900-9 (PMC12334603; doi:10.1038/s41598-025-13900-9)

Western blotting of C57BL/6j mice

SNAIL (29 KDa) Beta-actin (42 kDa)


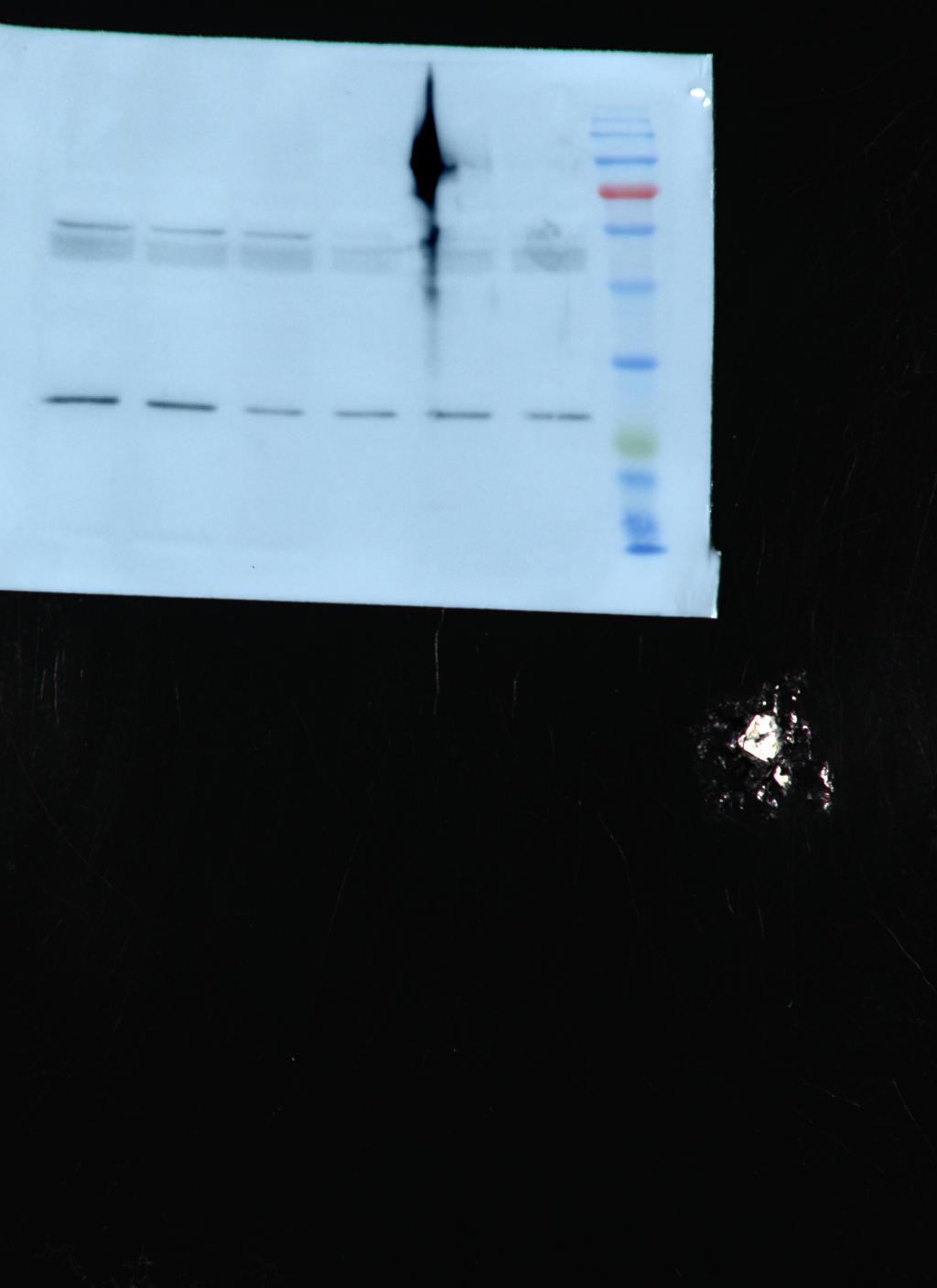

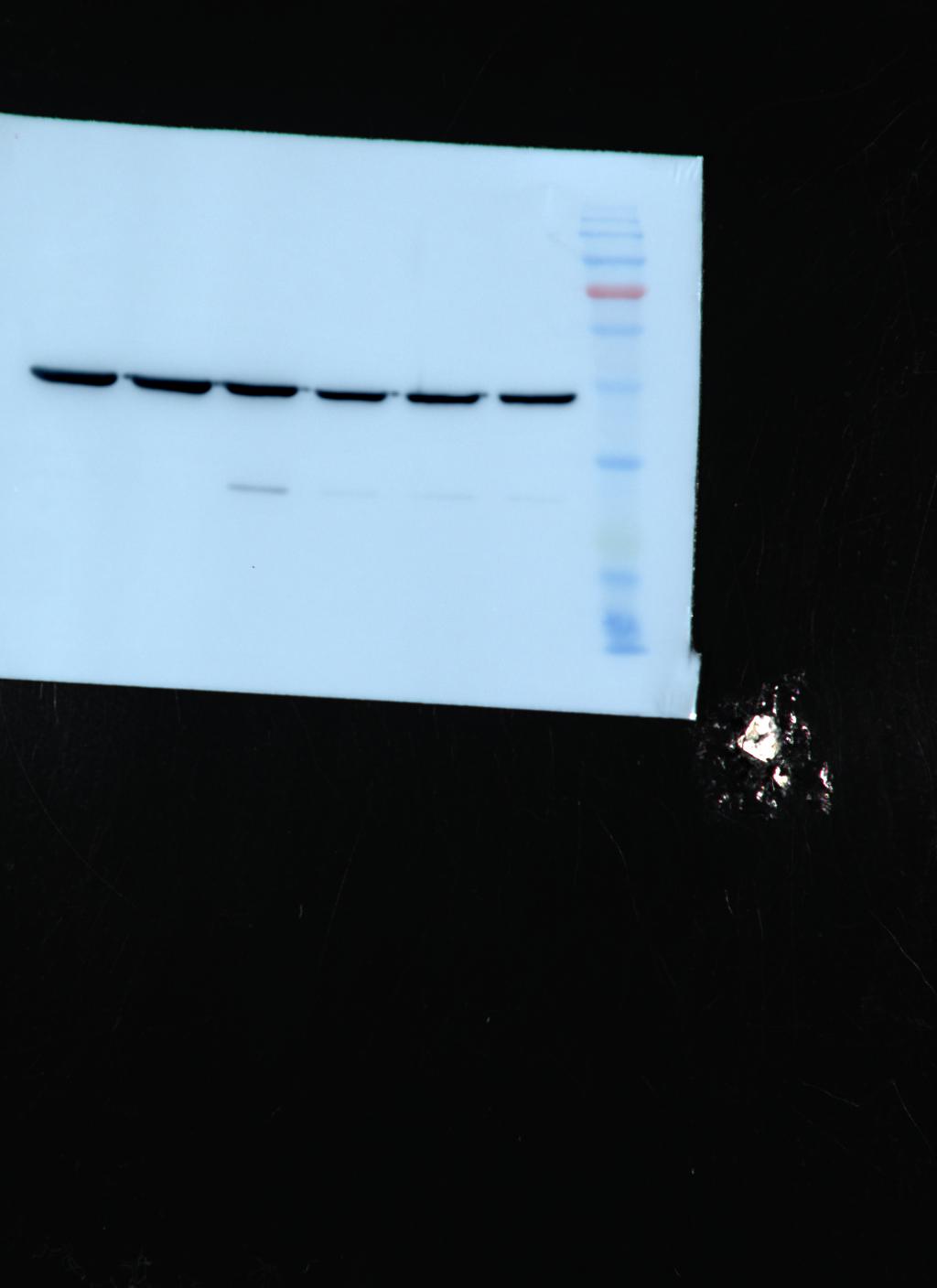


Vimentin (57 KDa) Beta-actin (42kDa)


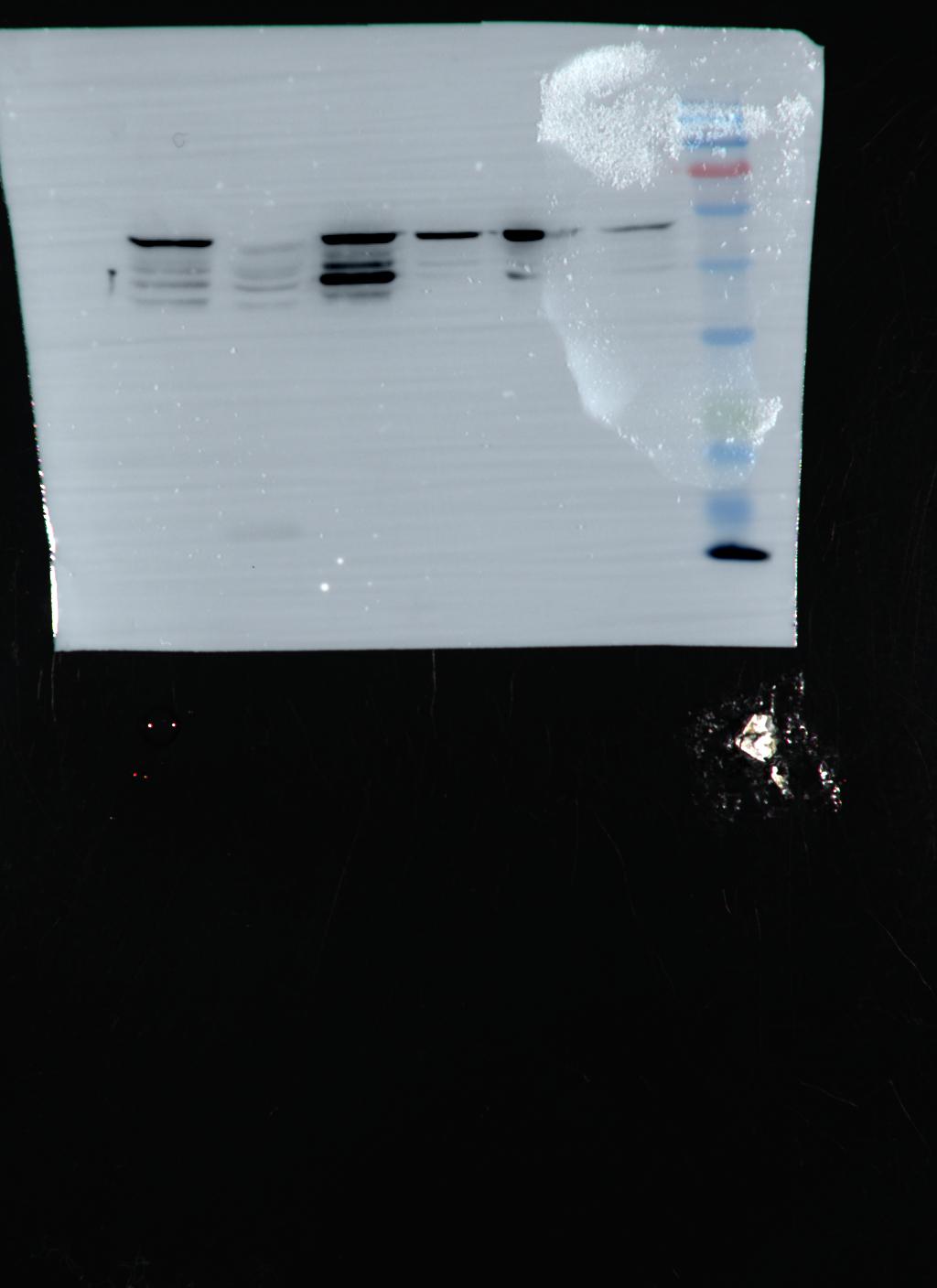

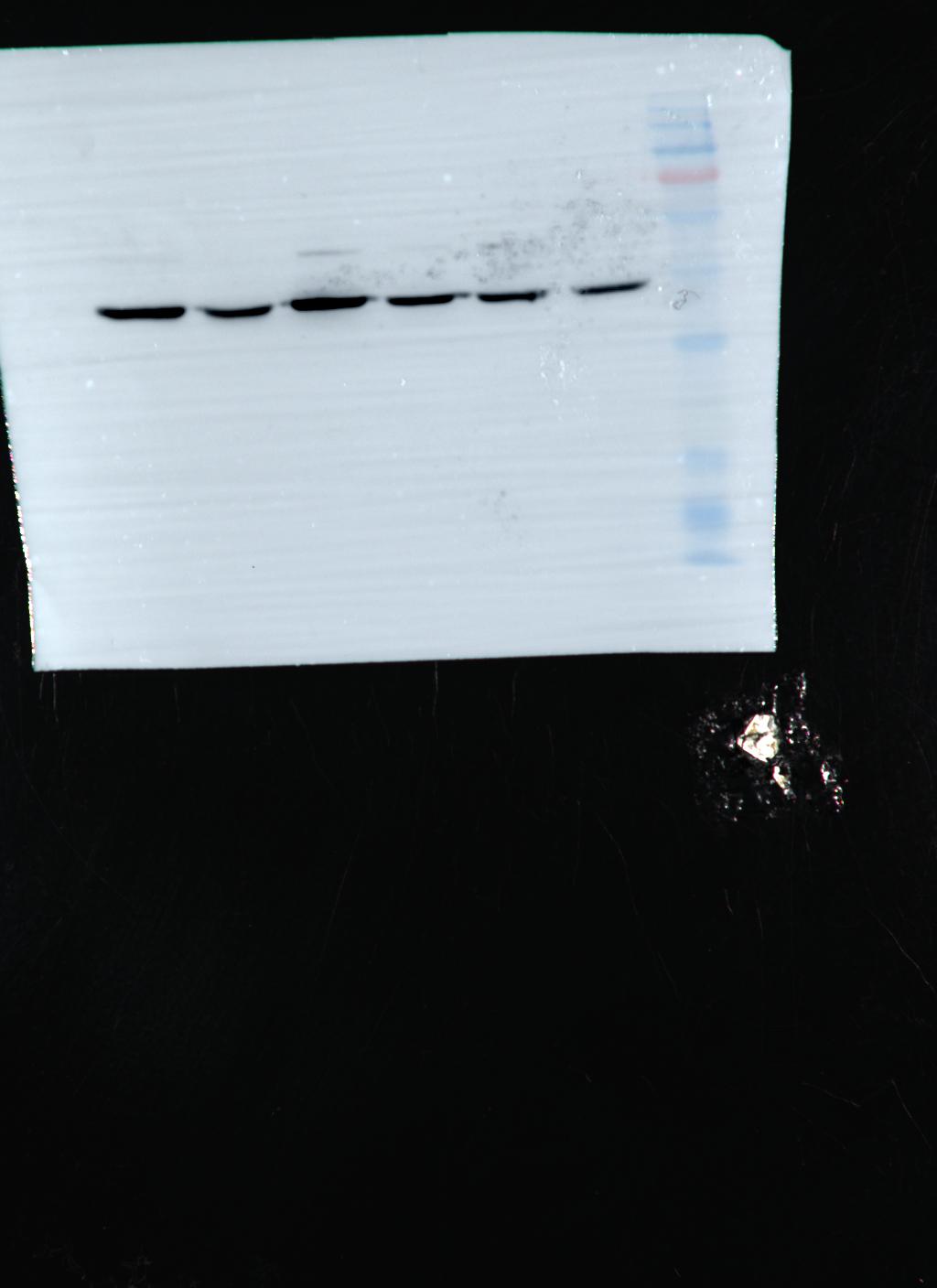


Cytokeratin (68 KDa) Beta-actin (42KDa)


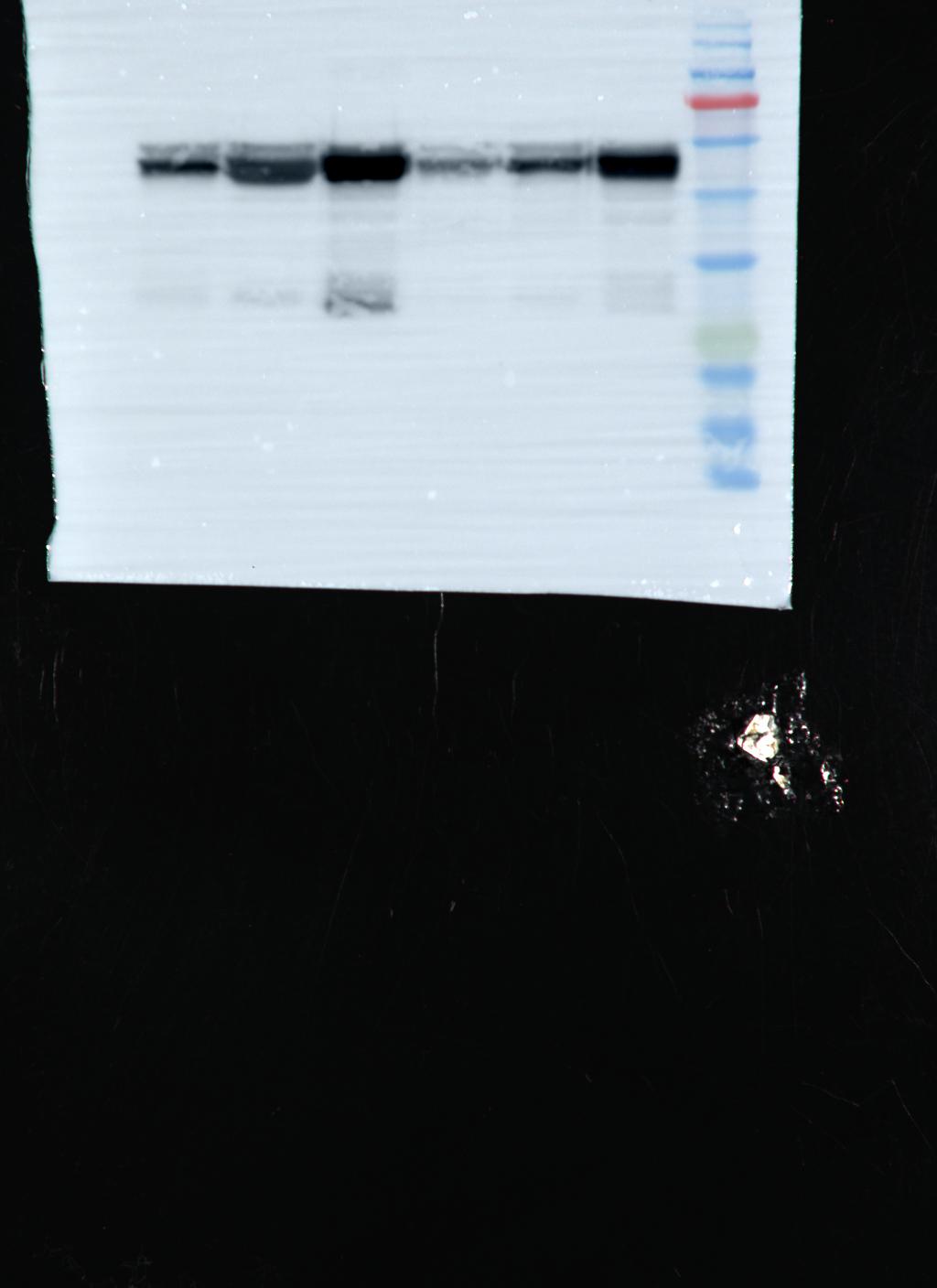

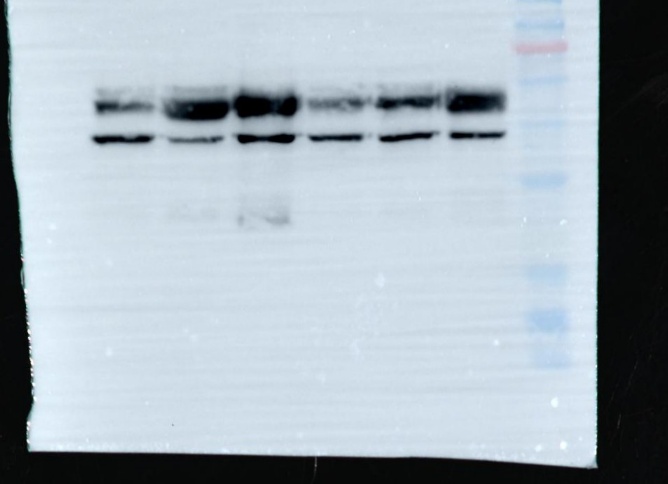

Supplement: Supplementary file 1 — Supplementary Material 1 [file 41598_2025_13900_MOESM1_ESM.docx]
